# European Union’s Public Fishing Access Agreements in Developing Countries

**DOI:** 10.1371/journal.pone.0079899

**Published:** 2013-11-27

**Authors:** Frédéric Le Manach, Christian Chaboud, Duncan Copeland, Philippe Cury, Didier Gascuel, Kristin M. Kleisner, André Standing, U. Rashid Sumaila, Dirk Zeller, Daniel Pauly

**Affiliations:** 1 Sea Around Us Project, Fisheries Centre, University of British Columbia, Vancouver, British Columbia, Canada; 2 Unité Mixte de Recherche 212 Ecosystèmes Marins Exploités, Institut de Recherche pour le Développement, Sète, France; 3 Bureau of National Fisheries, Ministry of Agriculture, Monrovia, Liberia; 4 Unité Mixte de Recherche 985 Ecologie et Santé des Ecosystème, Université Européenne de Bretagne, Rennes, France; 5 TransparentSea, Nairobi, Kenya; 6 U4 Anti-Corruption Resource Centre, Chr. Michelsen Institute, Bergen, Norway; 7 Fisheries Economics Research Unit, Fisheries Centre, University of British Columbia, Vancouver, British Columbia, Canada; Hawaii Pacific University, United States of America

## Abstract

The imperative to increase seafood supply while dealing with its overfished local stocks has pushed the European Union (EU) and its Member States to fish in the Exclusive Economic Zones of other countries through various types of fishing agreements for decades. Although European public fishing agreements are commented on regularly and considered to be transparent, this is the first global and historical study on the fee regime that governs them. We find that the EU has subsidized these agreements at an average of 75% of their cost (financial contribution agreed upon in the agreements), while private European business interests paid the equivalent of 1.5% of the value of the fish that was eventually landed. This raises questions of fisheries benefit-sharing and resource-use equity that the EU has the potential to address during the nearly completed reform of its Common Fisheries Policy.

## Introduction

Although 50% of Atlantic and 80% of Mediterranean marine resources are estimated to be overfished in European waters [1]–[3], Europe’s seafood demand continues to rise. Around 23 kg of seafood are currently consumed every year by each European Union (EU) citizen, with an increase of 2% per year [4]–[5]. The question of how to satisfy this rising demand, while decreasing its domestic fishing effort to avoid the collapse of its own fisheries and limiting its dependence on imports, has been on the EU’s agenda since the 1950s. The answer was the expansion of the EU fishing fleet further offshore and southward, often into the waters of developing countries [6]–[8]. Currently, a significant part of the EU catch is realized by a subset of the EU fleet comprised of 700 vessels, of which more than half fish under access agreements in the Exclusive Economic Zones (EEZs) of other countries and the rest within the High Seas [9]–[11].

In 1982, the United Nations Convention on the Law of the Sea (UNCLOS) was adopted, and its Article 62 forced distant-water fishing countries interested in fishing more (such as member countries of the EU) to sign fishing access agreements with host countries having a ‘surplus’ of resource, if they wished to fish within their 200 nm EEZs [12]. This Article is, however, based on two ambiguous notions, i.e., (i) that the ‘maximum sustainable yield’ can be estimated for most stocks in question, which is often impossible in developing countries [13]–[15]; and (ii) that the ‘total catch’ of these countries is known, which has been demonstrated not to be the case in all countries so far examined [16]–[22]. Joint scientific committees and regional fisheries management organizations are currently responsible for assessing the health of exploited stocks and setting fishing limits for both coastal and pelagic species targeted under European public fishing access agreements. However, concerns have been raised about the efficiency of such committees and organizations, mainly due to their lack of accessible data and analytical capacity [13], [23]–[24]. Consequently, to date, the vast majority of EU public fishing access agreements (and probably most agreements with other distant-water fishing countries) do not mention any quotas, and at best, refer to a ‘limit of reference’ (which can be exceeded for an additional payment, without any links to management targets, such as MSY, or stock status). This creates a loophole that both distant-water fishing countries and host countries use to maximize either their catch or their rent, sometimes at the expense of the resource’s health [25]–[27]. Indeed, any surpluses for fleets fishing in developing countries are likely to be lower than assumed. This is the case for the agreement with Mauritania, for example, which includes fishing possibilities for sardinella (*Sardinella aurita*) and cephalopds (mainly *Octopus vulgaris*), two overexploited stocks which together represent more than 50% of the landings of local artisanal fisheries but for which surpluses no longer exist [28]–[30].

**Figure 1:**
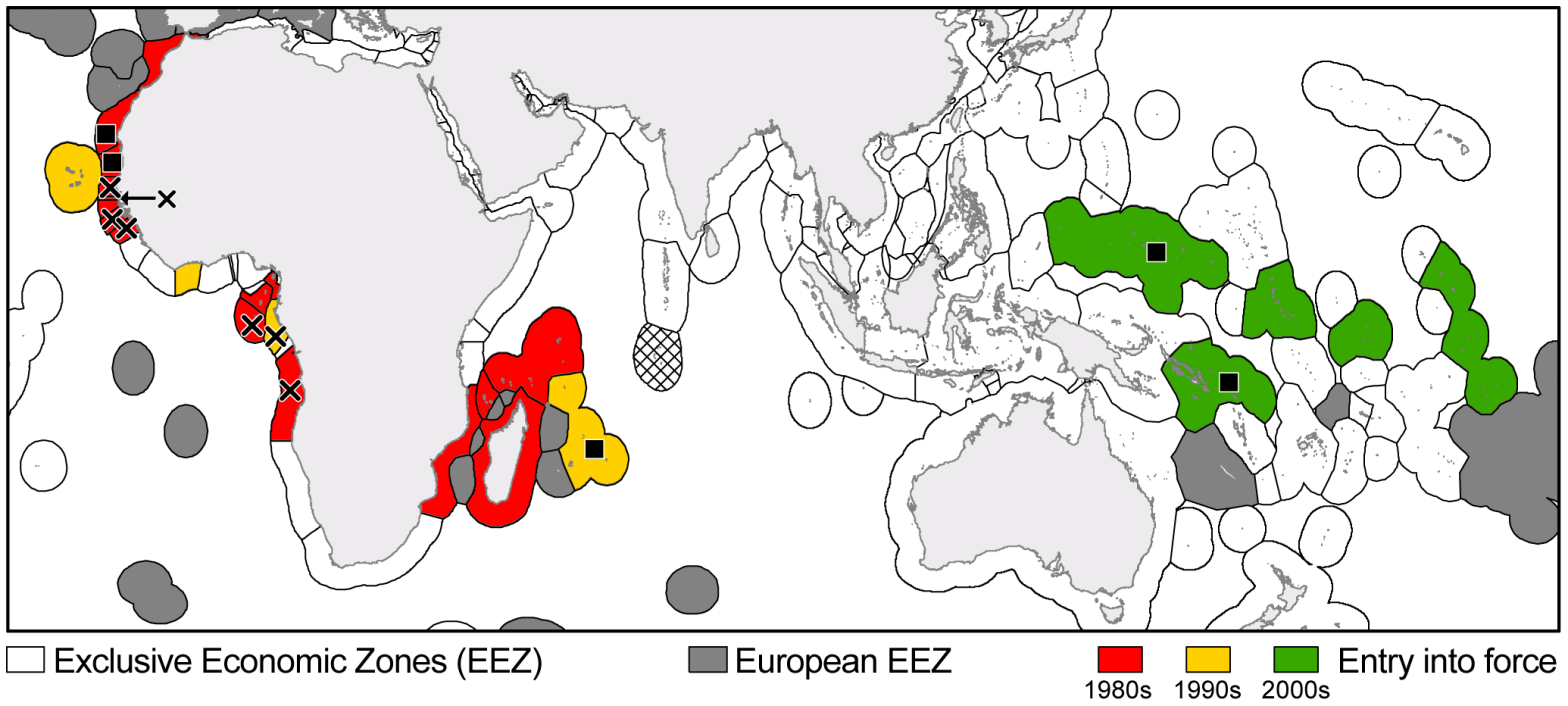
Extent of publicly funded EU fishing agreements with developing countries. Distribution of the twenty public agreements that the EU has signed with developing countries throughout Africa and Oceania in the 1980s (red), 1990s (yellow) and 2000s (green). EU vessels also have the possibility to freely fish in EU waters (represented by grey areas, including overseas territories with the exception of the Chagos Archipelago, in cross-hatch, which is now fully protected). Some of these agreements have been cancelled (Angola, Equatorial Guinea, Gabon, Gambia, Guinea-Bissau, Guinea, Senegal; highlighted with black crosses) or are currently being renegotiated (Mauritania, Mauritius, Micronesia, Morocco, Solomon Islands; highlighted with black squares). Situation as of December 2012.

Given UNCLOS, the EU created a strategic network of publicly funded fishing access agreements to govern access to valuable resources and support other private agreements made by its fishing industry with neighboring developing countries (**Figure 1**). Since the first public agreements between the EU and Guinea-Bissau and Senegal in 1980, which anticipated the adoption of UNCLOS, 18 other agreements have been signed throughout Africa and Oceania. These publicly funded fishing access agreements are designed to target either tuna and associated species, or coastal and demersal species (e.g., crustaceans, cephalopods, small pelagic fish, demersal fish). Most current agreements in West Africa (Cape Verde, Côte d’Ivoire, and São Tomé and Principe), in East Africa (except Mozambique until 2006), and Oceania strictly focus on tuna species (hereinafter termed ‘tuna agreements’). On the other hand, early agreements with West African countries covered mostly coastal and demersal species, typically with a small tuna component (hereinafter ‘mixed agreements’).

The distinction between tuna and mixed agreements is important because tuna agreements focus on pelagic species that seem to be less impacted by industrial fishing than demersal species [5]. Also, pelagic species are generally not heavily exploited by local fishers [6], [31], in contrast to the demersal species targeted by mixed agreements [28], [32]. Official EU documents (e.g., proposals [33] and working documents [34]) reveal it is likely that the EU will expand its network of tuna agreements (by signing new ones such as with the Cook Islands [35] or resuming previously cancelled ones, such as Gabon [36]), while continuing to phase out most of the demersal component of mixed agreements. The EU would, however, still allow the beneficiaries of current demersal licenses to create joint venture operations or reflag their fishing vessels [10], [37] outside the EU, effectively reducing the official capacity of the European fleet. Although beneficial as a means to add local value (e.g., job creation), this policy is deeply criticized as simply ‘exporting’ excess fishing effort to developing countries. Therefore, this is unlikely to end in a reduction of overfishing in many developing countries’ waters (especially for demersal resources [25], [27], [38]).

Many studies have focused on fishing agreements worldwide, and most have suggested that the fees received by host countries are low compared to the value of what is extracted, irrespective of the origin of the distant-water fishing country [6], [31], [39]–[48]. Recently, however, the situation seems to have improved in the Pacific [49]. Most of these studies are country- or region-specific, and usually cover a short period of time. For the European public fishing access agreements, these studies have had limited policy implications, although they are important at a local level, since they provided neither long-term trends nor a global analysis of these trends. Additionally, the breakdown of total fees paid by both the EU (i.e., subsidies) and the fishing industry has never been analyzed at a global scale. Here we provide a comprehensive analysis of the 33-year period covered by these publicly funded EU agreements (1980–2012). Focusing on the expanding tuna agreements, we analyze trends in each fee component (distant-water fishing country vs. host country, and then EU taxpayers vs. EU industry) to examine shortcomings related to these agreements and highlight historical trends, in order to provide ground for a sound reform of the European public fishing access agreements.

## Methods

For all twenty host countries that have been involved at some point in a public fishing access agreement with the EU, we retrieved all related official texts (namely, ‘council regulations/decisions’, ‘agreements’, ‘protocols’, and ‘information on the date of entry into force’) from the EU law database (http://eur-lex.europa.eu; also available in print). From these documents, we extracted the following data and information: (i) EEZ access fees and development aid (paid by EU taxpayers); (ii) fishing fees (paid by EU industry, either per tonne of fish or per unit of vessel capacity); (iii) fleet capacity; and (iv) specific ‘quotas’ and ‘limits of reference’ (both referred to as ‘quotas’ throughout this paper, although ‘limits of reference’ can be exceeded in exchange for additional payment), if available. This was done for each gear type or target species, country, and year (see **Table S1** and **References S1** for each country’s references).

This information was mostly collected from the ‘protocols’, although some of this information was only found in the ‘agreements’ for the earlier time-period. ‘Council regulations/decisions’ were mostly used to determine whether a renewed agreement was similar to its previous iteration, and ‘information on the date of entry into force’ documents were used to determine whether an agreement was active or not during any given year.

To produce a harmonized database, we then standardized the following units:

### Vessel Capacity

Gross Tonnage (GT) is the unit adopted by the International Convention on Tonnage Measurement of Ships in 1969 and entered into force in 1982. However, it seems that the use of this unit has not been enforced, as nearly all EU agreements used Gross Registered Tonnage (GRT). Therefore, we deemed a GT to GRT conversion (i.e., GRT is used in this paper as the common capacity unit) to be the best way to minimize our margin of error for the three countries whose fleet capacity was given in GT (Morocco from 2006 to present; Côte d’Ivoire from 2004 to present; and Mauritania from 1996 to present for non-tuna pelagic vessels, and then from 2006 to present for all other vessels). We are aware that GRT is not the best unit for discussing fishing capacity. For example, demersal trawlers are best defined by power of their engine, which drag their gear at the bottom of the ocean, while tuna vessels are better defined by the volume of their hold, which determines how long they can operate. However, GRT was the only unit we could use here to have a consistent fishing capacity unit, while limiting the number of assumptions and conversions.

Thus, we extracted records for which both GT and GRT were given (all countries) from the EU fleet registry (http://ec.europa.eu/fisheries/fleet), and kept records for (i) demersal gears (i.e., ‘beam trawls’, ‘bottom other trawls’, ‘bottom pair trawls’, ‘boat dredges’, ‘mechanized dredges’, ‘otter twin trawls’, ‘combined gillnets/trammelnets’, ‘set longlines’, and ‘set gillnets’; n = 25,423); and (ii) pelagic gears (i.e., ‘Danish seines’, ‘encircling gillnets’, ‘pair seines’, ‘purse seines’, ‘Scottish seines’; n = 3,175). We then performed a linear regression between GT and GRT for these two categories of gears (**Figure S1**; residuals were tested for normality in R and are presented in **Figure S2**), which we used to convert the GT given in the aforementioned agreements into GRT.

**Figure 2:**
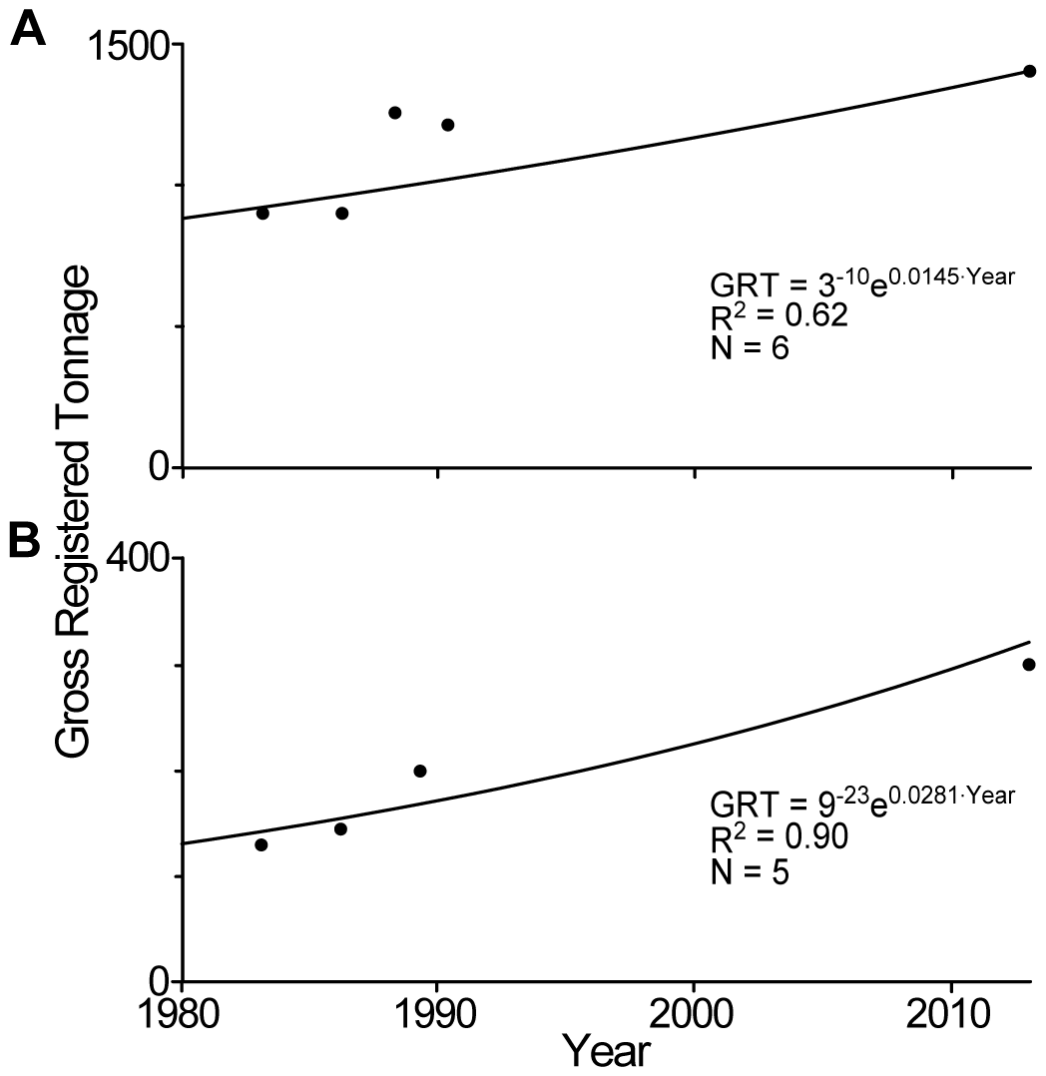
Trends of EU vessels’ Gross Registered Tonnage (GRT). Estimated mean annual capacity in GRT of A) tuna seiners and B) tuna liners (i.e., pole-and-line and longline vessels) deployed by the EU fleet from 1980 to 2012, suggesting that the mean GRT per vessel increases by 1.4% and 2.8% annually, respectively.

Furthermore, most tuna vessel capacities were provided only in number of vessels. To convert these numbers to GRT capacities, we used the correspondences between these two units provided for the 1980s in a handful of agreement documents (purse seiners: Guinea 1983 [50], Guinea-Bissau 1983 and 1986 [51]–[52], Senegal 1988 [53]–[54], Gambia 1990 [55]–[56] (we used the combination of the two consecutive protocols to estimate the mean GRT per purse seiner in the cases of Senegal and Gambia); ‘liners’, i.e., pole-and-line and longline vessels: Guinea 1983 [50], Guinea-Bissau 1983 and 1986 [51]–[52], Angola 1989 [57]). In order to get an additional anchor point for 2012, we calculated the geometric mean of the GRT per seiner, based on the list of seiners active in 2012 under EU agreements (provided by the *Observatoire Thonier*, Institut de Recherche pour le Développement, Sète, France), and GRT data available in the United Nations Food and Agriculture Organization’s ‘fishing vessels finder’ database (http://www.fao.org/figis/vrmf/finder/search/#stats). For liners, for which no such list was available, we instead computed the geometric mean of the tonnage of the 50 largest EU vessels (deemed to be representative of the EU industrial fleet; only 47 records available in the case of WCPFC) from each database of the three regional fisheries management organizations under which EU agreements operate (Atlantic Ocean: ICCAT; Indian Ocean: IOTC; Pacific Ocean: WCPFC). For each vessel type, we then fitted an exponential regression (**Figure 2**), which we used to convert numbers of vessels to GRT capacities (due to the low number of data points, we did not perform any analyses of the normality of the residuals).

Finally, non-tuna vessel capacity was also provided in numbers of vessels in seven cases, and we used correspondences given in other agreements for the same type of gear or species at the same period, and as much as possible, in the same region. A summary is provided in **Table S2**.

### Fees

In order to account for inflation over a given time-period, it is necessary to convert nominal values (i.e., the actual price in a given year) to real values that are comparable. All values in this paper are therefore given in real 2012 EUR (1 EUR = 1.28 USD), rather than nominal EUR.

From a European perspective, this conversion of the fees from nominal to real values required us to apply a Consumer Price Index (CPI) ‘deflator’ to nominal values:
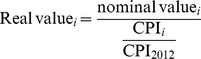
(1)where *i* represents the year for which the nominal value is converted into 2012 real value.

We extracted annual CPI data from the International Monetary Fund’s World Economic Outlook (http://www.imf.org/external/pubs/ft/weo/2012/02/weodata/index.aspx) for ‘Advanced Economies’. Note that this ‘Advanced Economies’ time-series exists for the entire 1980–2012 period, in contrast to Eurostat’s time-series of harmonized CPI for ‘Eurogroup’ (http://epp.eurostat.ec.europa.eu/portal/page/portal/hicp/data/database). However, these two time-series are very similar for the common 1992–2012 period, and thus we used the former.

Estimating the real value of the fees received by the host countries required that we performed one additional step. First, nominal EUR had to be converted to local currency units (LCU; exchange rates extracted from http://databank.worldbank.org; 2012 rates extracted from www.fxtop.com, up to October 2012) via US dollars, to which the host countries’ CPI was then applied, following **Equation 1**. However, host countries’ CPI time-series were not complete in the World Bank database, and we had to perform several interpolations to fill gaps, as presented in **Table S3** (final values are provided in **Dataset S1**).

This methodology is thought to represent an improvement on a preliminary estimate of Madagascar’s income [31], as we believe it estimates more accurately what host countries perceive they received over time by accounting more effectively for the inflation and money devaluation in host countries. It was developed following discussions with the European Directorate of Maritime Affairs and Fisheries (DG-MARE) and several colleagues. However, we note that although this new method provides a more accurate view of what a host country perceives it received, it does not change either the overall trend nor the discussion implied by such trends (at least in the case of Madagascar; see **Figure S3**).

Finally, global ex-vessel prices for the main species of tuna (yellowfin: *Thunnus albacares* and skipjack: *Katsuwonus pelamis*), as well as small pelagic fish, demersal fish species, shrimps and other crustaceans, and cephalopods were also extracted from a worldwide ex-vessel price database for the 1980–2006 period [58]–[59]. These ex-vessel prices were only collected for coastal countries of the European Union (Belgium, Bulgaria, Cyprus, Denmark, Estonia, Finland, France, Germany, Greece, Ireland, Italy, Latvia, Lithuania, Malta, the Netherlands, Poland, Portugal, Romania, Slovenia, Spain, Sweden, and the UK), as we assumed they were the ones mainly contributing to/benefiting from public EU fishing agreements. These ex-vessel prices, originally in nominal USD, were converted to 2012 EUR using annual USD-EUR exchange rates and Advanced Economies’ CPIs (from the World Bank and the International Monetary Fund, respectively). They are provided in **Dataset S1** and were used to estimate the ratio of ‘fees paid by the industry/gross revenue generated’.

We provide the final dataset in the appended Excel workbook (**Dataset S1**), as well as references for each country and year in **Table S1** and **References S1**. **Dataset S1** also includes comments to explain specific assumptions when the general methods presented here did not apply to a specific fleet, country and/or year.

## Results

### Agreements and Authorized Fishing Effort

**Figure 3:**
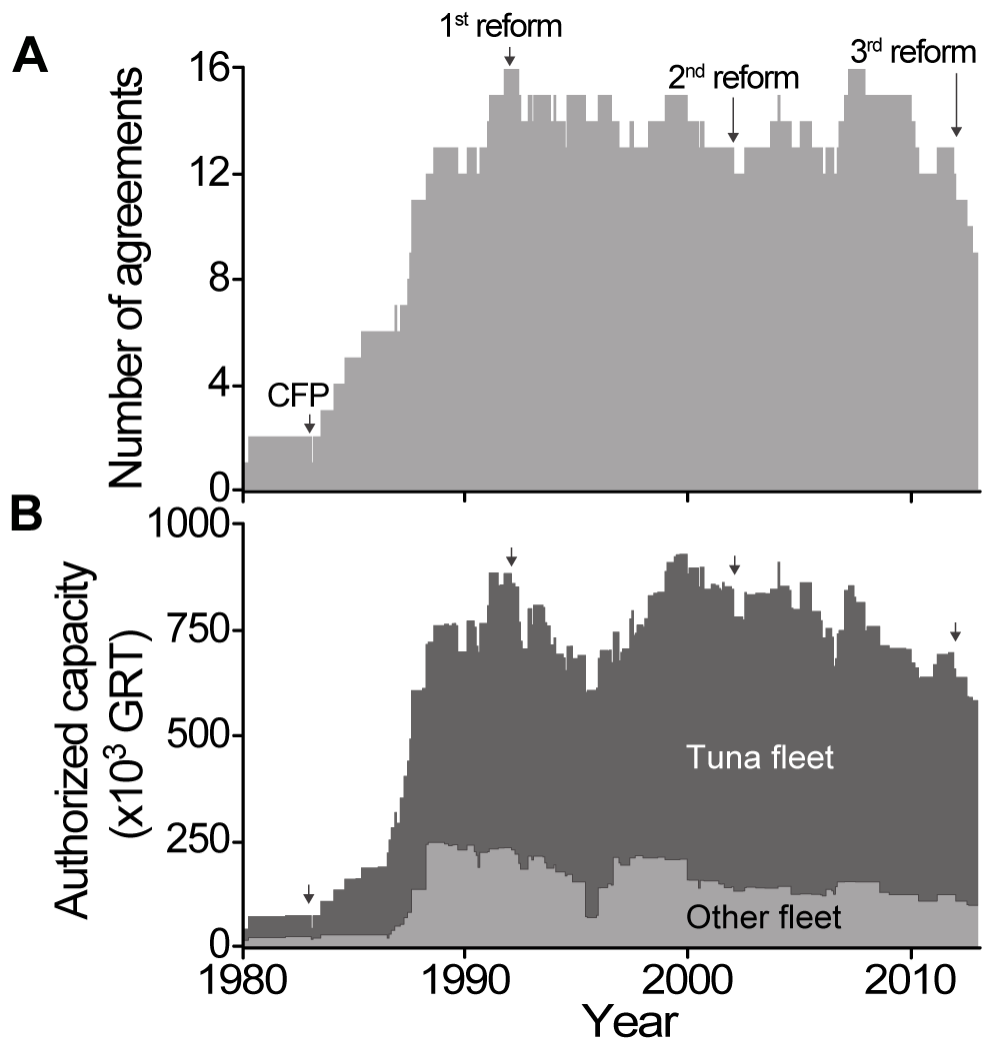
Number of agreements and authorized capacity. Trends of A) the number of EU public fishing agreements signed with developing countries in Africa and Oceania, and B) authorized capacity of the tuna fleet (dark grey) and other fleets (light grey; mostly demersal fishing, but also includes some non-tuna pelagic fishing with various types of lines and nets), from 1980 to 2012, by month. The implementation of the Common Fisheries Policy (CFP) and its three reforms (the third one being ongoing) are indicated by black arrows.

The number of European public fishing access agreements steeply increased after the implementation of the Common Fisheries Policy in 1983, from two during the 1980–1983 period (Senegal and Guinea-Bissau) to 16 in 1991 (**Figure 3A**). Subsequently, their numbers have mostly oscillated between 12 and 16. Since 2010, however, the total number of agreements steadily decreased to nine, notably because of difficulties to approve (e.g., Morocco, Micronesia) or renew some of them (e.g., Mauritius, Senegal), but also due to political instability (e.g., agreement with Guinea suspended in 2009). Their number is currently increasing again, as evidenced by the recent number of renewals and hints about new agreements being negotiated [33]–[36].

The changes in the number of agreements produced a pattern similar to that of the authorized fishing capacity, which rapidly increased from 40,000 GRT/year in the early 1980s to an average of just below 800,000 GRT/year over the 1990–2012 period (**Figure 3B**). However, overall fishing capacity declined since 2000, from over 900,000 GRT/year to slightly below 600,000 GRT/year in 2012, mainly due to the phasing out of mixed agreements, but also due to a more recent decline in the number of tuna agreements (or number of licenses available for each). Indeed, the fishing capacity of the fleet involved in mixed agreements continuously declined from around 250,000 GRT/year to below 100,000 GRT/year over the 1990–2012 period (**Figure 3B**). The fishing capacity of the tuna fleet seems to be increasing overall, although it peaked in the early 2000s at around 750,000 GRT/year and then decreased to slightly below 500,000 GRT in 2012.

### EU Subsidies

**Figure 4:**
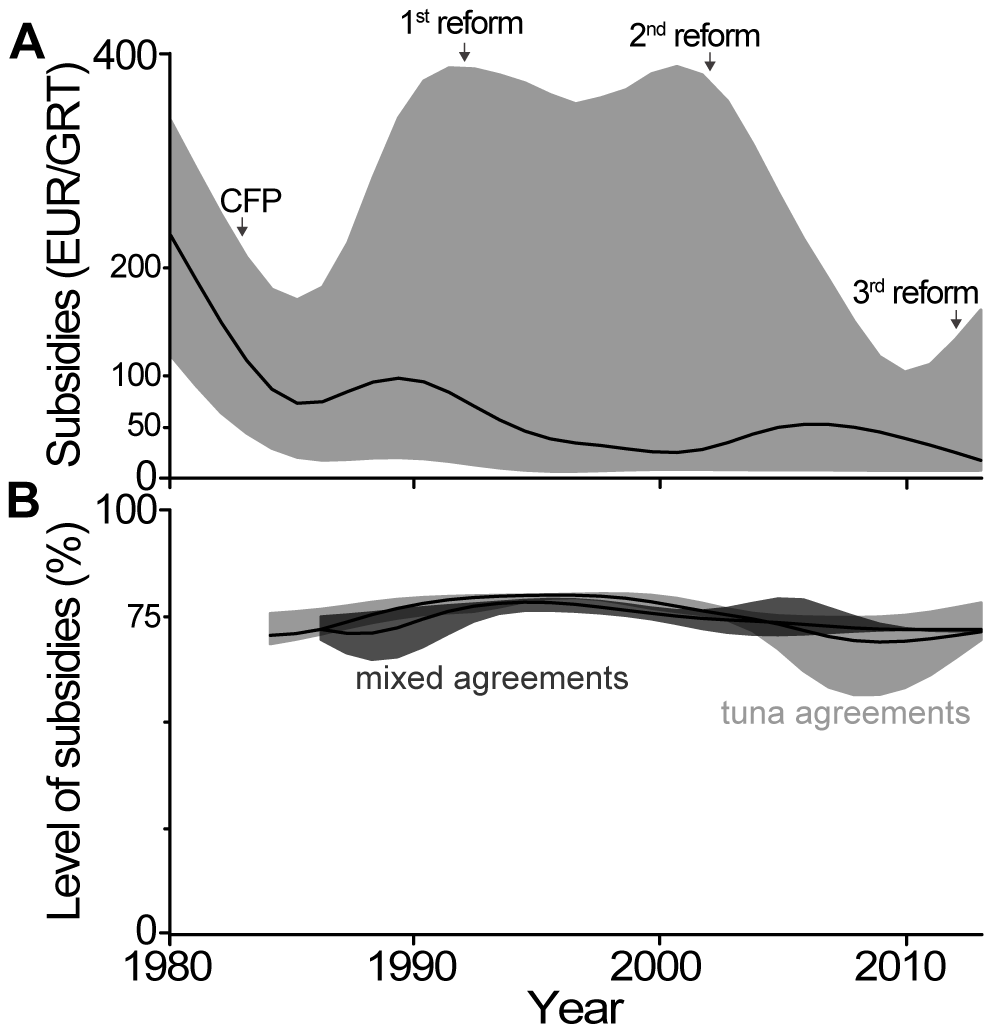
Level of public subsidies in EU agreements. Trend of A) the subsidies paid by the EU taxpayers per unit of capacity (2012 EUR/GRT/year) for all agreements; and B) the level of subsidies for both mixed (dark grey) and tuna (light grey) agreements. Panel B) only includes agreements for which there were tuna quotas. In both panels, the solid lines represent the median, while the colored areas represent the limit beyond which a point is considered to be an outlier [data points below quartile_1_−1.5*(quartile_3_ – quartile_2_), or above quartile_3_+1.5*(quartile_3_ – quartile_2_). The ‘smooth.spline’ function in R was used [82], with a smoothing window ‘spar’ set to 0.5]. Panel A is based on 397 ‘country/year’ datapoints, while panel B is based on 157 ‘country/year’ datapoints for the tuna agreements and 67 ‘country/year’ datapoints for the mixed agreements. The implementation of the Common Fisheries Policy (CFP) and its three reforms (the third one being ongoing) are indicated by black arrows.

Some countries (but certainly not Russia: http://ec.europa.eu/commission_2010-2014/damanaki/headlines/press-releases/2013/04/2010426_en.htm) or individuals may not consider the fees paid by the EU to be fisheries subsidies. However, they clearly confer a benefit to the fishing industry, thus meeting the World Trade Organization’s definition of subsidies [60]. These subsidies – paid by the EU taxpayers for each GRT allowed to fish in host countries’ waters – declined by half between 1980 and 1985 (from around 200 EUR/GRT/year [256 USD/GRT/year] to 100 EUR/GRT/year [128 USD/GRT/year]), then further declined to approximately 50 EUR/GRT/year (64 USD/GRT/year) in the 2000s (**Figure 4A**). The wide ribbon around the median (which corresponds to the limits beyond which any data point would be considered as an outlier, i.e., data points below quartile_1_−1.5*[quartile_3_ – quartile_2_], or above quartile_3_+1.5*[quartile_3_– quartile_2_]) illustrates the difference in the level of subsidies provided by each of the agreements. Indeed, this level of subsidies ranges from 11 EUR/GRT/year for Comoros to 1,816 EUR/GRT/year for Morocco (**Table 1**), and is above the 100 EUR/GRT/year threshold for only four other countries (i.e., Guinea-Bissau, Senegal, Angola, Mauritania; all involved in mixed agreements). **Figure S3** provides a breakdown of subsidies by country, and shows that the level of EU subsidies increased clearly over the entire time-period only for EU vessels fishing in Angola (agreement stopped in 2004), Guinea-Bissau and Mauritania. From the host countries’ perspective, the trend is fairly similar, as only Guinea-Bissau, Mauritania, São Tomé and Principe, and Senegal (agreement stopped in 2006) saw their financial income increase (although generally not over the last decade; **Figure S3**).

**Table 1:** Mean level of subsidies received by host countries over the period of their respective agreements, as well as mean fee paid by the industry per tonne of tuna over the 2008–2012 period.

Country	Subsidies (2012 EUR/GRT/year)	Tuna industry fee^a^ (2012 EUR/t)
Morocco	1,816	26.2
Mauritania	463	33.8
Angola	418	–
Senegal	247	–
Guinea-Bissau	159	31.2
Seychelles	86	35.0
Guinea	85	29.2
Solomon Islands	63	40.2
Kiribati	59	58.3
Mozambique	56	39.0
Micronesia	52	48.4
Equatorial Guinea	36	–
Gambia	29	–
Côte d’Ivoire	26	36.4
Gabon	23	36.7
Madagascar	23	36.4
Cape Verde	20	36.4
São Tomé and Principe	19	36.4
Mauritius	17	–
Comoros	11	43.8

a Liners usually pay lower fees than purse-seiners. Although purse-seiners catches are higher than that of liners, both gear types were given the same weight here.

### EU Tuna Industry Fees

**Figure 5:**
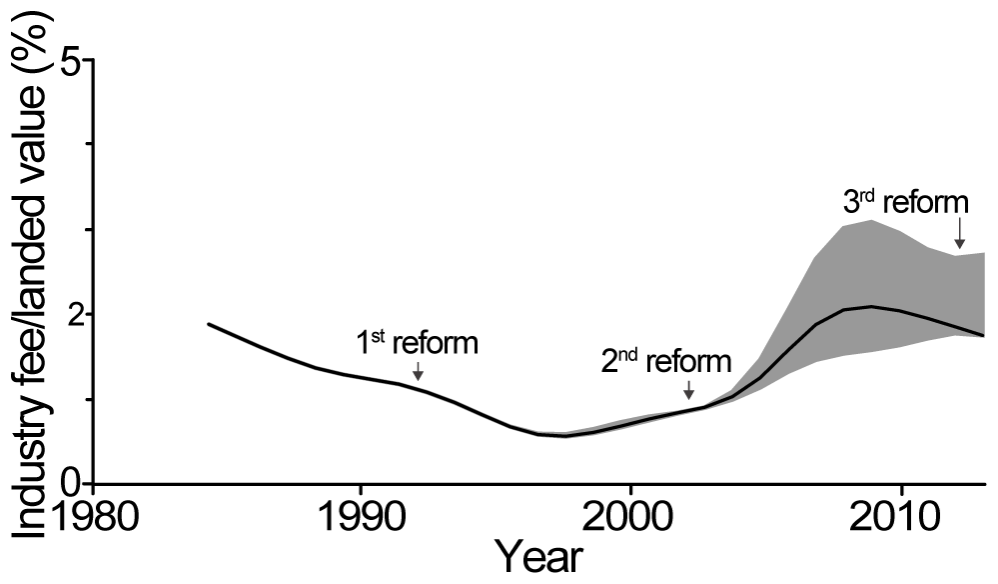
Cost of tuna agreements for the industry. Ratio of fees paid by the industry relative to landed value for the tuna component of all agreements. This figure only includes agreements for which there were tuna quotas. The solid line represents the median, while the grey area represents the limit beyond which a point is considered to be an outlier [data points below quartile_1_−1.5*(quartile_3_–quartile_2_), or above quartile_3_+1.5*(quartile_3_–quartile_2_). The ‘smooth.spline’ function in R was used [82], with a smoothing window ‘spar’ set to 0.5]. Note that for 2012, we considered the ex-vessel price of tuna to be 2,000 EUR/t, based on historical trends and various sources of information. This graph is based on 218 ‘country/year’ datapoints. Note that liners usually pay lower fees and also have lower catches than purse-seiners; however, both gear types were given the same weight here (the difference between weighted and non-weighted results was minimal). The implementation of the Common Fisheries Policy (CFP) and its three reforms (the third one being ongoing) are indicated by black arrows.

The comparison of the fees paid by the fishing industry to the landed value allowed us to estimate the revenue of the industry, in the context of increasingly widespread tuna agreements. The fees paid by the industry (i.e., approximately 25% of the total value of the agreements; see section above and **Figure 4B**) consistently represented less than 2% of its gross revenue (i.e., ‘ex-vessel price’ multiplied by ‘quota’). After an initial decrease from 2% in 1985 to around 0.8% in 1995, this percentage again increased to about 2% after the second reform of the Common Fisheries Policy (**Figure 5**). The wider ribbon around the median (which corresponds to the limits beyond which any data point would be considered as an outlier, i.e., data points below quartile_1_−1.5*[quartile_3_ – quartile_2_], or above quartile_3_+1.5*[quartile_3_ – quartile_2_]) over the past decade is mainly the result of higher fees negotiated in the Pacific, especially for Kiribati and Micronesia (**Table 1**).

### EU Demersal Industry Fees

For the demersal component of mixed agreements, it was not possible to globally estimate the historic industry’s fee/gross revenue ratio as computed for the tuna agreements (‘EU tuna industry fees’ section). Indeed, fees were provided in EUR/GRT/year and ex-vessel prices in EUR/t, while quotas were unavailable for virtually all countries and agreements. However, a detailed analysis of the country with the largest remaining mixed agreement, Mauritania, provides some insights. These results will therefore be seen as conservative, as the declared catch corresponds to the actual catch in the best-case scenario. However, there is often an un-reported or mis-reported component, therefore understating the benefits received by the fishing sector [42].

Over the 2008–2010 period, Mauritania received 80–95 million EUR/year of EU subsidies, which resulted in the export of 130–160,000 GRT/year in Mauritanian waters and total landings of 260–330,000 t/year (mainly small pelagics, but also octopuses, shrimps, and black hakes [61], [62]). On average, European taxpayers therefore paid 290 EUR/t of landed seafood. On the other hand, the industry paid between 9 EUR/t and 507 EUR/t of landed seafood, for small pelagics and crustaceans other than lobsters and crabs, respectively. In the newest agreement [63] - currently pending approval by the European Parliament - fees have steeply increased, and the EU industry expressed disquiet about this change, claiming this agreement was not worthwhile anymore [64]–[65].

We estimated the value of these catches (‘declared catch’ multiplied by ‘ex-vessel price’ = gross revenue) and calculated the ratio of ‘fees paid by the industry/gross revenue’. It appears that the industry has paid fees representing around 3.2% of their gross revenue from 2008 to 2010 (**Table 2**), which is almost twice as high as for tuna (see above).

**Table 2:** Baseline data and estimate of the ‘total fee/gross revenue’ ratio for the mixed agreement with Mauritania over the 2008–2010 period (details are shown in the appended Dataset S1; ‘Calculations Mauritania’ spreadsheet).

Name of fishing category		Ex-vesselprice (2012 EUR/t)	Declaredcatch (t/year)	Total fee(2012 EUR)^a^	Total fee/grossrevenue (%)
Official	In this paper				
Crustaceans except lobsters and crabs	Other crustaceans	12,814	3,358	1,601,850	4.0
Black hake	Black hake	4,041	3,974	290,924	1.8
Non-trawlers demersal vesselsfishing species other than black hake	Demersal vessels	5,183	1,841	192,310	2.0
Cephalopods	Demersal vessels	5,183	10,326	3,486,526	7.5
Pelagic vessels (freezer)	Pelagic vessels	803	272,440	5,314,779	2.4
Crabs	Other crustaceans	6,562	134	33,694	3.9
Pelagic vessels (wet)	Pelagic vessels	803	4,384	88,612	1.1

a ‘Total fee’ was calculated by multiplying the fee per unit of fishing capacity (in 2012 EUR/GT) by the amount of capacity (GT) reported to have been used in each fishing category [61].

## Discussion

Overall, the subsidies spent by taxpayers to grant the EU fishing industry the access to waters of host countries represent approximately 75% of the total value of the agreements for which such a ratio could be estimated (i.e., only agreements with quotas for their tuna component; **Figure 4B**). It can be argued, thus, that these subsidies allowed the maintenance of high fishing capacity by the EU in foreign waters. The high fishing effort that these subsidies generate is likely to have had detrimental effects on both the fish resources and fisheries development of host countries [25]–[27], [66]–[67]. Also, this high level of subsidization essentially means that the European consumer pays for the fish twice: once when it is caught, and again when it is bought. It could be argued that these agreements provide European consumers with cheaper fish. However, this is likely not the case, since European markets are supplied not only by the fleet fishing under such agreements [38], and prices of seafood are, to a large extent, globally harmonized in this market.

Our estimates of the fees paid by the industry relative to the fees paid by the European taxpayers (i.e., ∼25%) and the value of the industry fees relative to gross revenue (i.e., ∼1.5%) should be considered as conservative. First, the industry commonly does not use all annual fishing rights agreed upon in the agreements [68], while the EU usually still pays 100% of the EEZ access fees and development aid [69]–[70]. Therefore, it can be assumed that the industry commonly pays less than 25% of the total cost of these agreements. Second, there is increasingly widespread commercial use of bycatch and non-targeted species, such as sharks [23], [31] (exclusively targeted for their fins), which are not included in the industry’s fee. Therefore, the industry likely pays less than 1.5% of the overall landed value. The industry may argue here that they often pay licenses for fishing rights they do not use, but we believe that the overall impact on the global fee is low. Furthermore, as noted above, there is also often an un-reported or mis-reported component that understates the benefit of the industry (e.g., in the mid-2000s for the agreement with the Seychelles [71]). Furthermore, current partnership agreements only refer to the major targeted species. Thus, these committees and organizations do not focus on associated species or catches that are either discarded, or increasingly targeted when valuable. In some cases, bycatch species may even constitute an important or major part of total landings, often including overexploited or threatened resources. This is the case for many Mauritanian demersal fish stocks exploited as bycatch by Spanish shrimp trawlers, but not considered in the agreements and not accounted for in the financial compensation [28]–[29], [61], [72]. Based on recently collected evidence (e.g., records of Mozambican catches landed in South Africa [http://transparentsea.co/images/8/8a/Durban_landings.pdf] and European Commission data for Madagascar and Mozambique [http://transparentsea.co/images/d/dc/DG-MARE_sharks_lettre.pdf]), it appears that such free ‘side activities’ have become very important for some operators in the Indian Ocean, where Spanish longliners mostly target sharks [31], [73]–[74]. This targeted bycatch therefore represents an important part of the industry’s gross revenue and must be accounted for during the negotiation of financial compensation.

Although there are operating costs that the industry must cover (e.g., wages, fuel, taxes), it is expected that the net benefit accrued to the industry represents a sizeable part of the gross revenue, hence there is some room to raise the industry fishing fees. To assess what would be a ‘fair’ fee is a political decision requiring better understanding of the profits made by fishing firms operating under these access agreements. However, because account books are kept private, it is currently extremely difficult to estimate the industry’s net benefits, thus indirect methods via (global) fishing cost [75] and ex-vessel price databases [58]–[59] would be required. Interestingly, a senior representative of the French tuna fleet recently publicly acknowledged that the fee paid by the industry is low and that it would be reasonable to set it at up to 7% of the landed value [76]–[77]. This is a goal that host countries may consider right away. In approaching this issue, host countries in West Africa and the Indian Ocean could learn from the South Pacific Tuna Treaty, in which host countries are organized into a cartel-like organization [39], giving them more leverage to negotiate higher fishing fees. It is worth noting that Indian Ocean countries engaged in the current discussion about future regional allocation of quotas are heading towards adopting such a system. Removing subsidies and increasing benefit-sharing may also help ensure that the fishing fleet stays at a more sustainable size.

Overall, while one can argue that the amount host countries receive is not always negligible, EU agreements have clearly benefited the fishing industry more than the host countries. This discrepancy raises concerns of equitability and contradicts stated EU development goals, which suggests benefits from these agreements should be shared between both parties, not mainly directed towards private EU interests [34]. The current situation is a result of the ‘value for money’ requirement by the Court of Auditors [70], and therefore EU fishing agreements are business agreements above anything else. Harmonizing regulations among the various Directorate Generals would allow the creation of strengthened partnership [38], which could in turn help to ensure that host countries are less inclined to replace EU agreements with more opaque private business agreements or joint ventures, or agreements with countries that are less transparent or accountable in their agreements and negotiations (such as China [44]). Examples of this behaviour can be found, for example, in Mauritania [78]–[79] and Senegal [80]. Note that joint ventures can be perceived by host countries as more beneficial than foreign fishing access agreements, because they generate more local value, for example, through the construction of local processing plants and the creation of associated jobs [47]. It could be argued whether or not it would be in the interest of the EU to directly subsidize the development of a domestic fleet or joint ventures, which will in turn negatively impact the fishing opportunities offered to the EU distant fleet.

## Conclusion

Over the past few years, the EU has reiterated its wish to fish more sustainably and equitably [34], but also to expand its network of tuna agreements with African, Caribbean and Pacific countries. Sizeable improvements have already been achieved since the first agreements, for example, through clauses related to monitoring, local processing, and employment of local crew. Most of these improvements occurred with the shift from ‘fishing agreements’ to ‘fisheries partnership agreements’ in 2004 [38], [81]. However, much remains to be done with the creation of ‘sustainable fisheries agreements’ in the context of the 2012–2014 reform of the Common Fisheries Policy. If the EU intends to honor its stated goals, it needs to ensure that its fishing agreements (and subsequent EU fleet behaviour) are equitable, fair, enforced, and do not jeopardize the health of fish resources, or artificially channel benefits towards EU industrial beneficiaries.

During the remaining months of 2013 and in early 2014, the EU will determine its fishing policy for the forthcoming decade through the reform of its Common Fisheries Policy, and we hope that it will act in accordance with its stated public goals. The EU has the potential to become a global leader in equitable and sustainable fishing, especially given its existing attempts to move in this direction through the elimination of mixed agreements and the addition of various beneficial clauses in its remaining agreements. However, there are numerous advances that would need to be made to ensure a more balanced and equitable arrangement between the EU and the host countries. We hope that this study helps to clarify the rationale for taking such steps.

## Supporting Information

Figure S1 **Correlation between Gross Registered Tonnage (GRT) and Gross Tonnage (GT).** Linear regression of the records collected from the EU vessel registry (http://ec.europa.eu/fisheries/fleet) for which tonnages in both GT and GRT were available. Panel A) corresponds to demersal gears (n = 25,423), while B) represents pelagic gears (n = 3,175). In both cases, there is a strong correlation between the two parameters GT and GRT (r^2^ = 0.94 for demersal gears, and r^2^ = 0.98 for demersal gears). Note that neither GRT nor GT are normally distributed, as there are fewer vessels with higher tonnages (i.e., the distribution of the tonnage is skewed towards 0). The analysis of normality of the residuals is presented in **Figure S2**.
(TIF)

Figure S2 **Analysis of the residuals of the linear regression presented in Figure S1.** Panel A) and B) correspond to demersal gears (n = 25,423), while C) and D) represent pelagic gears (n = 3,175). Panels A) and C) show that the accuracy of our GRT estimates diminish when GT increases, and the right panels show that the residuals have a distribution that is relatively normal (perfect normality would be obtained if all points were on the horizontal line). Although there are a few residuals that deviate from normality, they did not impact our parameter estimation, given the large sample size (n).
(TIF)

Figure S3 **Trend of EU subsidies by country.** Country-breakdown of normalized EU subsidies in real value, seen from the EU’s perspective (thick line; 2012 EUR/GRT) and that of the host countries (thin line; 2012 LCU/GRT). The Pearson correlation coefficient *r* between these two time-series is given for each country (*indicates p-value<0.001; the sample size for each country, i.e., the number of years for which there was an agreement, is provided in **Table S1**). For 13 out of 20 countries, there is a statistically significant, strong correlation (*r*>0.70) between the subsidies paid by the EU taxpayers (in 2012 EUR/GRT) and what the host countries perceive they received (in 2012 LCU/GRT). The EU has clearly decreased its subsidies to 11 of these countries (at least in the last decade), which also translated into decreasing income for the host countries.
(TIF)

Table S1 References for official documents used to collect data, and summary of the correspondences between names in each of these official texts and the database.
(DOCX)

Table S2 Correspondence between the number of vessels and GRT capacities for non-tuna vessels.
(DOCX)

Table S3 Assumptions made to fill the gaps in the CPI time-series.
(DOCX)

Dataset S1 Data extracted from the 1980–2012 European Union’s fishing access agreements, aggregated by gear type or species group. The first tab of the dataset (‘Data’) contains the fishing effort, the quotas/limits of reference, the industrial fees, and the EU subsidies, for each month a given country had an active agreement with the European Union. The ‘Data’ tab also contains various parameters used for our calculations, such as CPI and exchange rates. The following tabs contain the pivot tables used to aggregate the data shown in Figures 4A–B, 5, and S3, as well as Table 2.
(XLSX)

References S1 References used in **Table S1** and **Table S2**.
(DOCX)
