# Supplementary figures and images for: European Union’s Public Fishing Access Agreements in Developing Countries
Source: PLoS One. 2013 Nov 27;8(11):e79899. doi: 10.1371/journal.pone.0079899 (PMC3842348; doi:10.1371/journal.pone.0079899)

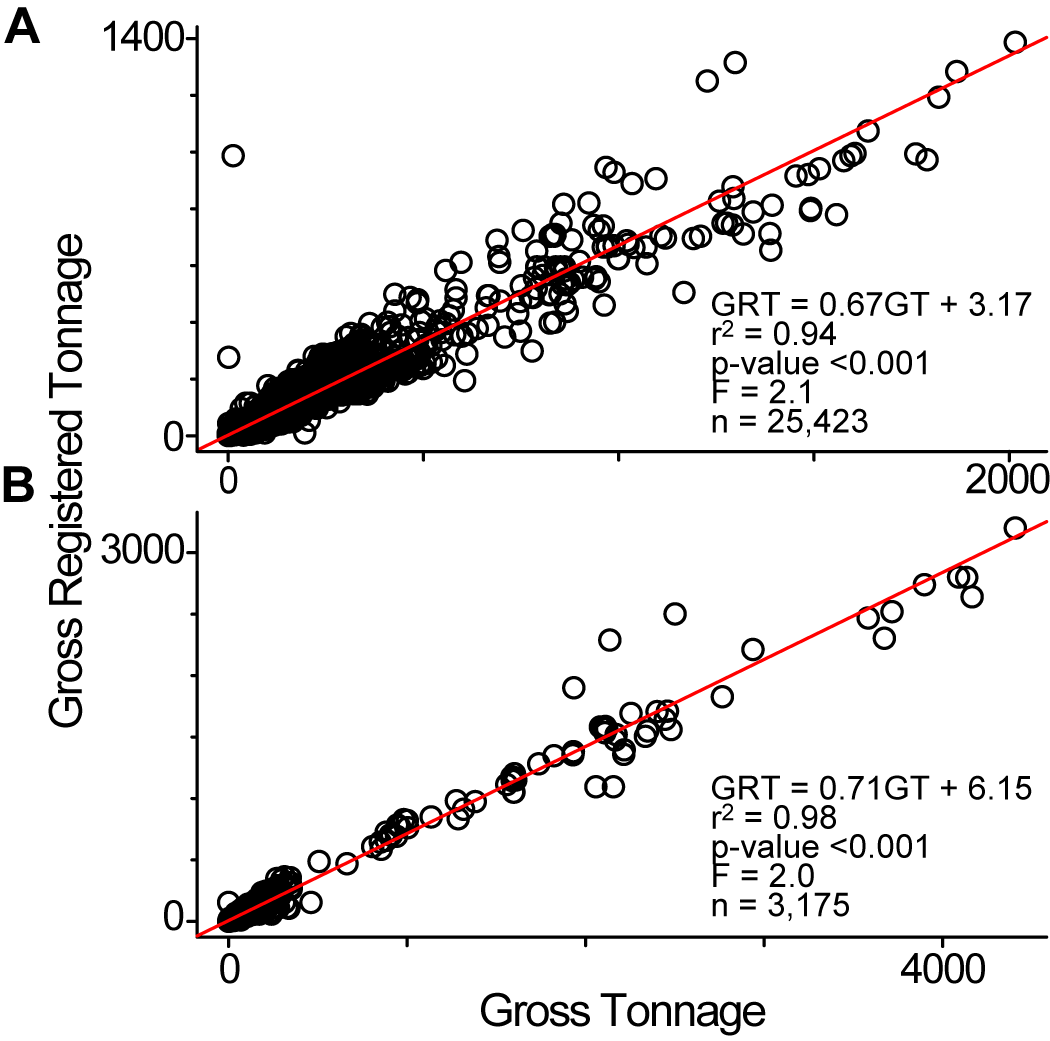

Supplement: Figure S1 — Correlation between Gross Registered Tonnage (GRT) and Gross Tonnage (GT). Linear regression of the records collected from the EU vessel registry (http://ec.europa.eu/fisheries/fleet) for which tonnages in both GT and GRT were available. Panel A) corresponds to demersal gears (n = 25,423), while B) represents pelagic gears (n = 3,175). In both cases, there is a strong correlation between the two parameters GT and GRT (r2 = 0.94 for demersal gears, and r2 = 0.98 for demersal gears). Note that neither GRT nor GT are normally distributed, as there are fewer vessels with higher tonnages (i.e., the distribution of the tonnage is skewed towards 0). The analysis of normality of the residuals is presented in Figure S2. (TIF) [file pone.0079899.s001.tif]

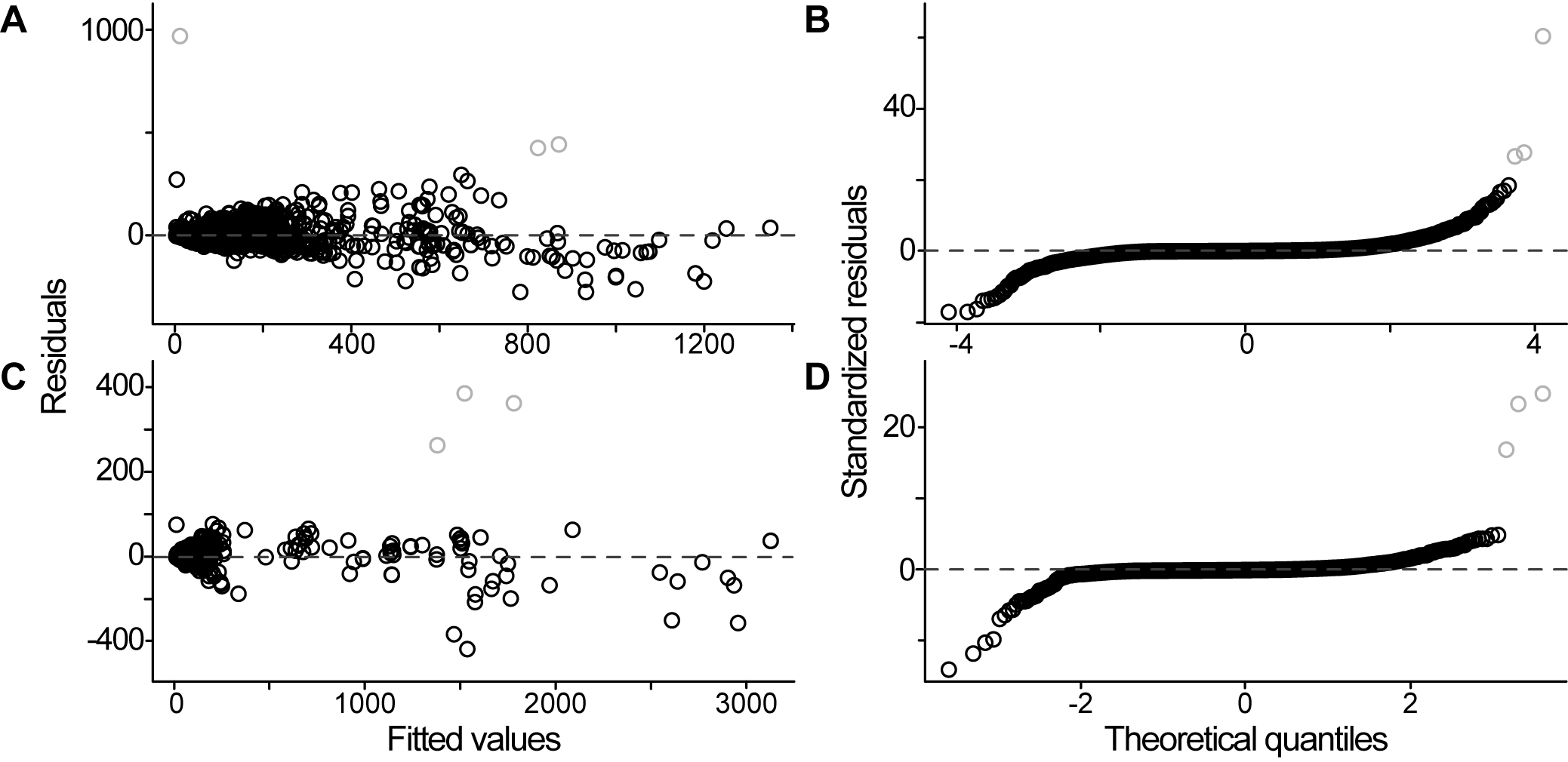

Supplement: Figure S2 — Analysis of the residuals of the linear regression presented in Figure S1. Panel A) and B) correspond to demersal gears (n = 25,423), while C) and D) represent pelagic gears (n = 3,175). Panels A) and C) show that the accuracy of our GRT estimates diminish when GT increases, and the right panels show that the residuals have a distribution that is relatively normal (perfect normality would be obtained if all points were on the horizontal line). Although there are a few residuals that deviate from normality, they did not impact our parameter estimation, given the large sample size (n). (TIF) [file pone.0079899.s002.tif]

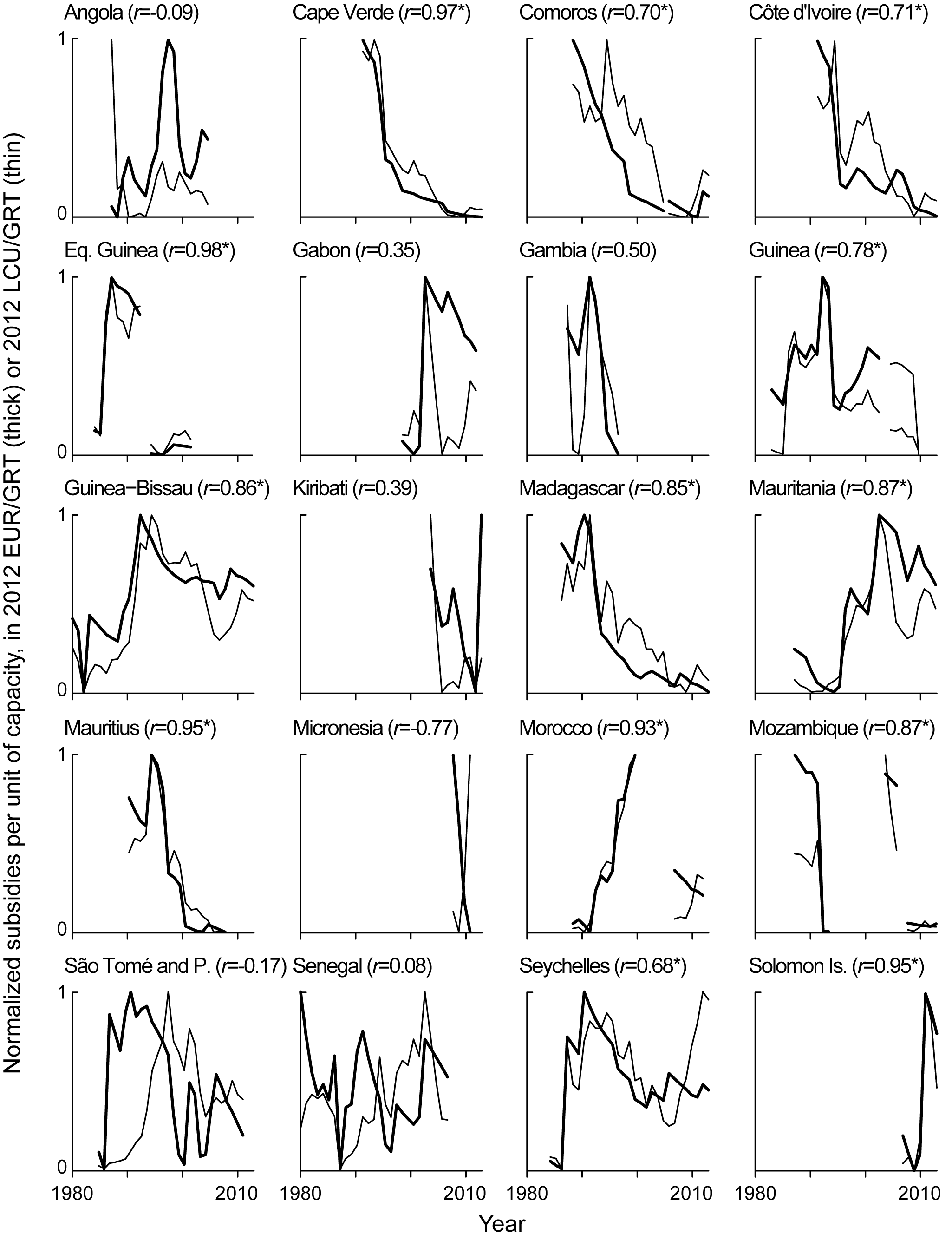

Supplement: Figure S3 — Trend of EU subsidies by country. Country-breakdown of normalized EU subsidies in real value, seen from the EU’s perspective (thick line; 2012 EUR/GRT) and that of the host countries (thin line; 2012 LCU/GRT). The Pearson correlation coefficient r between these two time-series is given for each country (*indicates p-value<0.001; the sample size for each country, i.e., the number of years for which there was an agreement, is provided in Table S1). For 13 out of 20 countries, there is a statistically significant, strong correlation (r>0.70) between the subsidies paid by the EU taxpayers (in 2012 EUR/GRT) and what the host countries perceive they received (in 2012 LCU/GRT). The EU has clearly decreased its subsidies to 11 of these countries (at least in the last decade), which also translated into decreasing income for the host countries. (TIF) [file pone.0079899.s003.tif]
