# Supplementary material for: European Union’s Public Fishing Access Agreements in Developing Countries
Source: PLoS One. 2013 Nov 27;8(11):e79899. doi: 10.1371/journal.pone.0079899 (PMC3842348; doi:10.1371/journal.pone.0079899)
Supplement: Table S1 — References for official documents used to collect data, and summary of the correspondences between names in each of these official texts and the database. (DOCX) [file pone.0079899.s004.docx]

| Table S1. References for official documents used to collect data, and summary of the correspondences between names in each of these official texts and the database. | | | | |
| --- | --- | --- | --- | --- |
| Country | Period | References ^a^ | Names in | |
|  |  |  | Official texts | Dataset S1 |
| Angola | May 1987 to May 1989 | [1,2] | Shrimp trawlers | Shrimp |
|  |  |  | Ocean going tuna freezers | Tuna seiners |
|  | May 1989 to May 1990 | [3] | Shrimp trawlers | Shrimp |
|  |  |  | Ocean going tuna freezers | Tuna seiners |
|  |  |  | Demersal trawlers | Demersal vessels |
|  |  |  | Surface longliners | Tuna liners |
|  | May 1990 to May 1992 | [4] | Shrimp vessels | Shrimp |
|  |  |  | Ocean going tuna freezers | Tuna seiners |
|  |  |  | Wet tuna boats | Tuna liners |
|  |  |  | Demersal trawlers | Demersal vessels |
|  | May 1992 to May 1994 | [5] | Shrimp vessels | Shrimp |
|  |  |  | Demersal trawlers | Demersal vessels |
|  |  |  | Freezer tuna seiners | Tuna seiners |
|  |  |  | Surface longliners | Tuna liners |
|  | May 1994 to May 1996 | [6] | Shrimp vessels | Shrimp |
|  |  |  | Demersal trawlers | Demersal vessels |
|  |  |  | Bottom set longlines, fixed gillnets | Demersal vessels |
|  |  |  | Freezer tuna seiners | Tuna seiners |
|  |  |  | Surface longliners | Tuna liners |
|  | May 1996 to May 2000 | [7,8] | Shrimp vessels | Shrimp |
|  |  |  | Demersal trawlers | Demersal vessels |
|  |  |  | Bottom set longlines, fixed gillnets | Demersal vessels |
|  |  |  | Freezer tuna seiners | Tuna seiners |
|  |  |  | Surface longliners | Tuna liners |
|  |  |  | Vessels for pelagic species | Pelagic vessels |
|  | May 2000 to August 2004 | [9-11] | Shrimp vessels | Shrimp |
|  |  |  | Demersal vessels | Demersal vessels |
|  |  |  | Bottom set longlines, fixed gillnets | Demersal vessels |
|  |  |  | Freezer tuna seiners | Tuna seiners |
|  |  |  | Surface longliners | Tuna liners |
|  |  |  | Vessels for pelagic species | Pelagic vessels |
| Cape Verde | September 1991 to September 1997 | [12-14] | Freezer tuna seiners | Tuna seiners |
|  |  |  | Pole-and-line tuna vessels | Tuna liners |
|  |  |  | Surface longliners | Tuna liners |
|  |  |  | Bottom longliners | Demersal vessels |
|  |  |  | Cephalopod fishing vessels | Demersal vessels |
|  | September 1997 to September 2000 | [15] | Freezer tuna seiners | Tuna seiners |
|  |  |  | Pole-and-line tuna vessels | Tuna liners |
|  |  |  | Surface longliners | Tuna liners |
|  |  |  | Bottom longliners | Demersal vessels |
|  | July 2001 to June 2005 | [16,17] | Tuna seiners | Tuna seiners |
|  |  |  | Pole-and-line tuna vessels | Tuna liners |
|  |  |  | Surface longliners | Tuna liners |
|  |  |  | Bottom longliners | Demersal vessels |
|  | September 2006 to December 2012 | [18,19] | Tuna seiners | Tuna seiners |
|  |  |  | Pole-and-line tuna vessels | Tuna liners |
|  |  |  | Surface longliners | Tuna liners |
| Comoros | July 1988 to July 1997 | [20-23] | Ocean going freezer tuna vessels | Tuna seiners |
|  | March 1998 to February 2004 | [24,25] | Freezer tuna seiners | Tuna seiners |
|  |  |  | Surface longliners | Tuna liners |
|  | January 2005 to December 2012 | [26,27] | Tuna seiners | Tuna seiners |
|  |  |  | Surface longliners | Tuna liners |
| Côte d’Ivoire | July 1991 to June 2007 | [28-34] | Freezer demersal trawlers (fish, invert.) | Demersal vessels |
|  |  |  | Surface longliners | Tuna liners |
|  |  |  | Pole-and-line tuna vessels | Tuna liners |
|  |  |  | Tuna seiners | Tuna seiners |
|  | July 2007 to December 2012 | [35] | Tuna seiners | Tuna seiners |
|  |  |  | Surface longliners | Tuna liners |
| Equatorial Guinea | July 1984 to June 1986 | [36,37] | Ocean going freezer tuna boats | Tuna seiners |
|  | July 1986 to June 1989 | [38,39] | Freezer trawlers | Demersal vessels |
|  |  |  | Freezer tuna seiners | Tuna seiners |
|  |  |  | Pole-and-line tuna vessels | Tuna liners |
|  | July 1989 to June 1992 | [40] | Freezer trawlers | Demersal vessels |
|  |  |  | Tuna seiners | Tuna seiners |
|  |  |  | Surface longliners | Tuna liners |
|  | July 1994 to June 2001 | [41-43] | Freezer tuna seiners | Tuna seiners |
|  |  |  | Pole-and-line tuna vessels | Tuna liners |
|  |  |  | Surface longliners | Tuna liners |
| Gabon | December 1998 to November 2001 | [44] | Freezer tuna seiners | Tuna seiners |
|  |  |  | Surface longliners | Tuna liners |
|  | December 2001 to November 2005 | [45] | Demersal freezer trawlers (crustaceans, cephalopods) | Demersal vessels |
|  |  |  | Freezer tuna seiners | Tuna seiners |
|  |  |  | Surface longliners | Tuna liners |
|  | December 2005 to November 2011 | [46] | Freezer tuna seiners | Tuna seiners |
|  |  |  | Surface longliners | Tuna liners |
| Gambia | July 1987 to June 1990 | [47,48] | Freezer seiners tuna vessels | Tuna seiners |
|  |  |  | Pole and line tuna vessels | Tuna liners |
|  |  |  | Long liners | Tuna liners |
|  |  |  | Fresh fish trawlers | Demersal vessels |
|  |  |  | Other fresh fish vessels (crustaceans) | Demersal vessels |
|  |  |  | Shrimp freezer trawlers | Shrimp |
|  |  |  | Other freezer trawlers | Demersal vessels |
|  | July 1990 to June 1993 | [49] | Freezer seiners tuna vessels | Tuna seiners |
|  |  |  | Pole and line tuna vessels | Tuna liners |
|  |  |  | Surface longliners | Tuna liners |
|  |  |  | Fresh fish trawlers | Demersal vessels |
|  |  |  | Other fresh fish vessels (crustaceans) | Demersal vessels |
|  |  |  | Shrimp freezer trawlers | Shrimp |
|  |  |  | Other freezer trawlers | Demersal vessels |
|  | July 1993 to June 1996 | [50] | Freezer seiners tuna vessels | Tuna seiners |
|  |  |  | Pole and line tuna vessels | Tuna liners |
|  |  |  | Fresh fish trawlers | Demersal vessels |
|  |  |  | Shrimp freezer trawlers | Shrimp |
|  |  |  | Other freezer trawlers | Demersal vessels |
| Guinea-Bissau | March 1980 to December 1982 | [51-54] | Bottom trawlers | Demersal vessels |
|  |  |  | Freezer tuna boats | Tuna seiners |
|  | June 1983 to May 1986 | [55] | Bottom trawlers | Demersal vessels |
|  |  |  | Freezer tuna vessels | Tuna seiners |
|  |  |  | Wet pole-and-line tuna vessels | Tuna liners |
|  | June 1986 to May 1989 | [56,57] | Trawlers | Demersal vessels |
|  |  |  | Freezer tuna seiners | Tuna seiners |
|  |  |  | Wet pole-and-line tuna vessels | Tuna liners |
|  |  |  | Longliners | Tuna liners |
|  | June 1989 to May 2012 | [58-65] | Freezer shrimp trawlers | Shrimp |
|  |  |  | Freezer fin fish and cephalopod trawlers | Demersal vessels |
|  |  |  | Freezer tuna seiners | Tuna seiners |
|  |  |  | Pole-and-line tuna vessels | Tuna liners |
|  |  |  | Surface longliners | Tuna liners |
| Guinea | February 1983 to August 1986 ^b^ | [66-68] | Trawlers | Demersal vessels |
|  |  |  | Shrimp boats | Shrimp |
|  |  |  | Ocean-going freezer tuna boats | Tuna seiners |
|  |  |  | Wet tuna liners | Tuna liners |
|  | August 1986 to July 1989 | [69,70] | Trawlers | Demersal vessels |
|  |  |  | Freezer tuna seiners | Tuna seiners |
|  |  |  | Wet pole-and-line tuna vessels | Tuna liners |
|  |  |  | Longliners | Tuna liners |
|  | January 1990 to December 1997 | [71-74] | Trawlers | Demersal vessels |
|  |  |  | Freezer tuna seiners | Tuna seiners |
|  |  |  | Pole-and-line tuna vessels | Tuna liners |
|  |  |  | Surface longliners | Tuna liners |
|  | January 1998 to December 2008 ^b^ | [75-78] | Fin-fish and cephalopod trawlers | Demersal vessels |
|  |  |  | Shrimp trawlers | Shrimp |
|  |  |  | Freezer tuna seiners | Tuna seiners |
|  |  |  | Pole-and-line tuna vessels | Tuna liners |
|  |  |  | Surface longliners | Tuna liners |
|  | January 2009 to December 2012 | [79] | Freezer tuna seiners | Tuna seiners |
|  |  |  | Pole-and-line tuna vessels | Tuna liners |
| Kiribati | September 2003 to December 2012 | [80-82] | Purse seine vessels | Tuna seiners |
|  |  |  | Long-liners | Tuna liners |
| Madagascar | June 1986 to May 1989 | [83-85] | Ocean-going freezer tuna boats | Tuna seiners |
|  |  |  | Crabs and deep-water shrimps vessels | Other crustaceans |
|  | June 1989 to May 1992 | [86] | Ocean-going freezer tuna boats | Tuna seiners |
|  |  |  | Deep water crustacean vessels | Other crustaceans |
|  | June 1992 to December 2006 | [87-91] | Freezer tuna seiners | Tuna seiners |
|  |  |  | Surface longliners | Tuna liners |
|  | January 2007 to December 2012 | [92] | Freezer tuna seiners | Tuna seiners |
|  |  |  | Surface longliners | Tuna liners |
|  |  |  | Line or longline bottom-fishing vessels | Demersal vessels |
| Mauritania | July 1987 to June 1990 | [93,94] | Lobster boats | Other crustaceans |
|  |  |  | Shrimp boats | Shrimp |
|  |  |  | Black hake trawlers | Black hake |
|  |  |  | Pelagic seiners | Pelagic vessels |
|  |  |  | Non-industrial pelagic seiners | Non-industrial vessels |
|  |  |  | Pole-and-line tuna vessels | Tuna liners |
|  |  |  | Surface longliners | Tuna liners |
|  | August 1990 to July 1993 | [95] | Crustacean fishing vessels (except lobsters) | Other crustaceans |
|  |  |  | Black hake trawlers and bottom longliners | Black hake |
|  |  |  | Pelagic trawlers and seiners | Pelagic vessels |
|  |  |  | Lobsters pot vessels | Other crustaceans |
|  |  |  | Pole-and-line tuna vessels | Tuna liners |
|  |  |  | Surface longliners | Tuna liners |
|  |  |  | Freezer tuna seiners | Tuna seiners |
|  | August 1993 to July 1996 | [96,97] | Crustacean fishing vessels (except crawfish) | Other crustaceans |
|  |  |  | Black hake trawlers and bottom longliners | Black hake |
|  |  |  | Fixed gillnet, longliner, line demersal vessels | Demersal vessels |
|  |  |  | Deepwater demersal trawlers | Demersal vessels |
|  |  |  | Crawfish pot vessels | Other crustaceans |
|  |  |  | Pole-and-line tuna vessels | Tuna liners |
|  |  |  | Surface longliners | Tuna liners |
|  |  |  | Freezer tuna seiners | Tuna seiners |
|  |  |  | Cephalopod fishing vessels | Demersal vessels |
|  | August 1996 to July 2006 | [98,99] | Crustacean fishing vessels (except crawfish) | Other crustaceans |
|  |  |  | Black hake trawlers and bottom longliners | Black hake |
|  |  |  | Fixed gillnet, longliner, line demersal vessels | Demersal vessels |
|  |  |  | Deepwater demersal trawlers | Demersal vessels |
|  |  |  | Crawfish pot vessels | Other crustaceans |
|  |  |  | Pole-and-line tuna vessels | Tuna liners |
|  |  |  | Surface longliners | Tuna liners |
|  |  |  | Freezer tuna seiners | Tuna seiners |
|  |  |  | Cephalopod fishing vessels | Demersal vessels |
|  |  |  | Pelagic freezer trawlers | Pelagic vessels |
|  | August 2006 to July 2008 | [100] | Crustacean fishing vessels (except crawfish and crab) | Other crustaceans |
|  |  |  | Crab pot vessels | Other crustaceans |
|  |  |  | Crawfish pot vessels | Other crustaceans |
|  |  |  | Black hake trawlers and bottom longliners | Black hake |
|  |  |  | Fixed gillnet, longliner, line demersal vessels | Demersal vessels |
|  |  |  | Demersal trawlers | Demersal vessels |
|  |  |  | Cephalopod fishing vessels | Demersal vessels |
|  |  |  | Tuna seiners | Tuna seiners |
|  |  |  | Pole-and-line tuna vessels | Tuna liners |
|  |  |  | Surface longliners | Tuna liners |
|  |  |  | Pelagic freezer trawlers | Pelagic vessels |
|  |  |  | Non-freezer pelagic vessels | Pelagic vessels |
|  | August 2008 to July 2012 | [101] | Crustacean fishing vessels (except lobster and crab) | Other crustaceans |
|  |  |  | Crab pot vessels | Other crustaceans |
|  |  |  | Lobster pot vessels | Other crustaceans |
|  |  |  | Black hake trawlers and bottom longliners | Black hake |
|  |  |  | Fixed gillnet, longliner, line demersal vessels | Demersal vessels |
|  |  |  | Demersal trawlers | Demersal vessels |
|  |  |  | Cephalopod fishing vessels | Demersal vessels |
|  |  |  | Tuna seiners | Tuna seiners |
|  |  |  | Pole-and-line tuna vessels | Tuna liners |
|  |  |  | Surface longliners | Tuna liners |
|  |  |  | Pelagic freezer trawlers | Pelagic vessels |
|  |  |  | Non-freezer pelagic vessels | Pelagic vessels |
|  | August 2012 to December 2012 ^c^ | [102] | Crustacean fishing vessels (except lobster and crab) | Other crustaceans |
|  |  |  | Black hake trawlers and bottom longliners | Black hake |
|  |  |  | Fixed gillnet, longliner, line demersal vessels | Demersal vessels |
|  |  |  | Crab pot vessels | Other crustaceans |
|  |  |  | Tuna seiners | Tuna seiners |
|  |  |  | Pole-and-line tuna vessels | Tuna liners |
|  |  |  | Surface longliners | Tuna liners |
|  |  |  | Pelagic freezer trawlers | Pelagic vessels |
|  |  |  | Non-freezer pelagic vessels | Pelagic vessels |
|  |  |  | Cephalopod fishing vessels | Demersal vessels |
| Mauritius | December 1990 to November 1993 | [103-105] | Ocean-going tuna seiners | Tuna seiners |
|  |  |  | Line fishing vessels | Demersal vessels |
|  |  |  | Crustacean fishing vessels | Other crustaceans |
|  | December 1993 to November 1999 | [106,107] | Ocean-going tuna seiners | Tuna seiners |
|  |  |  | Line fishing vessels | Lines |
|  | December 1999 to November 2007 | [108-110] | Tuna seiners | Tuna seiners |
|  |  |  | Surface longliners | Tuna liners |
|  |  |  | Line fishing vessels | Lines |
| Micronesia | March 2007 to December 2012 | [111,112] | Purse seine vessels | Tuna seiners |
|  |  |  | Long-liners | Tuna liners |
| Morocco | March 1988 to April 1992 | [113-116] | Trawlers | Demersal vessels |
|  |  |  | Seiners | Pelagic vessels |
|  |  |  | Longline, trammel net, gillnet | Demersal vessels |
|  |  |  | Vessel gathering sponges | Demersal vessels |
|  |  |  | Non-industrial vessels | Non-industrial vessels |
|  |  |  | Fresh cephalopod vessels | Demersal vessels |
|  |  |  | Freezer cephalopod vessels | Demersal vessels |
|  |  |  | Black hake trawlers | Black hake |
|  |  |  | Demersal trawlers | Demersal vessels |
|  |  |  | Pelagic trawlers | Pelagic vessels |
|  |  |  | Lobster pot vessels | Other crustaceans |
|  |  |  | Shrimp and prawn vessels | Shrimp |
|  |  |  | Pole-and-line tuna vessels | Tuna liners |
|  |  |  | Frostfish longliners | Demersal vessels |
|  | May 1992 to April 1995 | [117,118] | Shrimp trawlers | Shrimp |
|  |  |  | Others trawlers | Demersal vessels |
|  |  |  | Seine nets | Pelagic vessels |
|  |  |  | Sponge vessels | Demersal vessels |
|  |  |  | Longline, trammel net, fixed gillnet | Demersal vessels |
|  |  |  | Non-commercial vessels | Non-industrial vessels |
|  |  |  | Cephalopod vessels | Demersal vessels |
|  |  |  | Black hake trawlers | Black hake |
|  |  |  | Demersal trawlers | Demersal vessels |
|  |  |  | Pelagic trawlers | Pelagic vessels |
|  |  |  | Pole-and-line and seine nets with live bait | Tuna liners |
|  | December 1995 to November 1999 | [119,120] | Cephalopod vessels | Demersal vessels |
|  |  |  | Shrimp trawlers | Shrimp |
|  |  |  | Longline, trammel net, fixed gillnet | Demersal vessels |
|  |  |  | Seine nets | Pelagic vessels |
|  |  |  | Small-scale vessels | Non-industrial vessels |
|  |  |  | Black hake trawlers | Black hake |
|  |  |  | Pelagic trawlers | Pelagic vessels |
|  |  |  | Sponge vessels | Demersal vessels |
|  |  |  | Tuna vessels | Tuna liners |
|  | March 2006 to December 2011 | [121,122] | Seine nets | Non-industrial vessels |
|  |  |  | Small-scale vessels | Non-industrial vessels |
|  |  |  | Pole-and-line tuna vessels and seine nets with live bait | Tuna liners |
|  |  |  | Bottom longliners, bottom trawls, fixed gillnet | Demersal vessels |
|  |  |  | Industrial pelagic vessels | Pelagic vessels |
| Mozambique | January 1987 to December 1989 | [123,124] | Deepwater shrimp vessels | Shrimp |
|  |  |  | Shrimp vessels | Shrimp |
|  |  |  | Ocean-going freezer tuna boats | Tuna seiners |
|  | January 1990 to December 1991 | [125,126] | Deepwater shrimp vessels | Shrimp |
|  |  |  | Shrimp vessels | Shrimp |
|  |  |  | Ocean-going tuna seiners | Tuna seiners |
|  | January 1992 to September 1993 | [127] | Ocean-going freezer tuna vessels | Tuna seiners |
|  | January 2003 to December 2005 | [128] | Deepwater shrimp demersal freezer trawlers | Shrimp |
|  |  |  | Freezer tuna seiners | Tuna seiners |
|  |  |  | Surface longliners | Tuna liners |
|  | January 2007 to December 2011 | [129] | Freezer tuna seiners | Tuna seiners |
|  |  |  | Surface longliners | Tuna liners |
|  | January 2012 to December 2012 | [130] | Ocean-going freezer tuna seiners | Tuna seiners |
|  |  |  | Surface longliners | Tuna liners |
| Sao Tome and Principe | April 1985 to October 1986 | [131-133] | Ocean-going freezer tuna boats | Tuna seiners |
|  | June 1987 to May 1993 | [134-136] | Freezer tuna seiners | Tuna seiners |
|  |  |  | Wet pole-and-line tuna vessels | Tuna liners |
|  | June 1993 to May 1996 | [137] | Freezer tuna seiners | Tuna seiners |
|  |  |  | Wet pole-and-line tuna vessels or surface longliners | Tuna liners |
|  | June 1996 to May 2006 | [138-141] | Freezer tuna seiners | Tuna seiners |
|  |  |  | Pole-and-line tuna vessels | Tuna liners |
|  |  |  | Surface longliners | Tuna liners |
|  | June 2006 to May 2010 | [142] | Freezer tuna seiners | Tuna seiners |
|  |  |  | Surface longliners | Tuna liners |
|  | June 2010 to December 2012 | [143] | Tuna seiners | Tuna seiners |
|  |  |  | Surface longliners | Tuna liners |
| Senegal | January 1980 to October 1981 | [144-146] | Tuna boats | Tuna seiners |
|  |  |  | Trawlers | Demersal vessels |
|  | November 1981 to December 1983 | [147,148] | Tuna boats | Tuna seiners |
|  |  |  | Shrimp trawlers | Shrimp |
|  |  |  | Fish trawlers | Demersal vessels |
|  | January 1984 to May 1986 | [149-152] | Tuna boats | Tuna seiners |
|  |  |  | Trawlers | Trawlers |
|  | October 1986 to February 1988 | [153] | Tuna boats | Tuna seiners |
|  |  |  | Shrimp wet trawlers | Shrimp |
|  |  |  | Shrimp freezer trawlers | Shrimp |
|  |  |  | Fish wet trawlers | Demersal vessels |
|  |  |  | Fish freezer trawlers | Demersal vessels |
|  | March 1988 to April 1990 | [154,155] | Shrimp wet demersal trawlers | Shrimp |
|  |  |  | Fish and cephalopods wet demersal trawlers | Demersal vessels |
|  |  |  | Ocean-going wet fish trawlers | Demersal vessels |
|  |  |  | Shrimp freezer demersal trawlers | Shrimp |
|  |  |  | Fish and cephalopods freezer demersal trawlers | Demersal vessels |
|  |  |  | Ocean-going shrimp freezer trawlers | Shrimp |
|  |  |  | Tuna vessels | Tuna liners |
|  |  |  | Freezer tuna seiners | Tuna seiners |
|  |  |  | Surface longliners | Tuna liners |
|  | May 1990 to April 1992 | [156] | Fish and cephalopods wet demersal trawlers | Demersal vessels |
|  |  |  | Ocean-going wet fish trawlers | Demersal vessels |
|  |  |  | Fish and cephalopods freezer demersal trawlers | Demersal vessels |
|  |  |  | Ocean-going shrimp freezer trawlers | Shrimp |
|  |  |  | Tuna vessels | Tuna liners |
|  |  |  | Freezer tuna seiners | Tuna seiners |
|  |  |  | Surface longliners | Tuna liners |
|  | October 1992 to September 1994 | [157] | Fish and cephalopods wet demersal trawlers | Demersal vessels |
|  |  |  | Ocean-going deepwater trawlers | Demersal vessels |
|  |  |  | Fish and cephalopods freezer demersal trawlers | Demersal vessels |
|  |  |  | Ocean-going shrimp freezer trawlers | Shrimp |
|  |  |  | Bottom longliners | Demersal vessels |
|  |  |  | Tuna canners | Tuna liners |
|  |  |  | Freezer tuna seiners | Tuna seiners |
|  |  |  | Surface longliners | Tuna liners |
|  | October 1994 to October 1996 | [158,159] | Fish and cephalopods wet demersal trawlers | Demersal vessels |
|  |  |  | Ocean-going deepwater trawlers | Demersal vessels |
|  |  |  | Fish and cephalopods freezer demersal trawlers | Demersal vessels |
|  |  |  | Ocean-going shrimp freezer trawlers | Shrimp |
|  |  |  | Pole-and-line tuna vessels | Tuna liners |
|  |  |  | Freezer tuna seiners | Tuna seiners |
|  |  |  | Surface longliners | Tuna liners |
|  | May 1997 to December 2001 | [160,161] | Fish and cephalopods demersal (freezer) trawlers | Demersal vessels |
|  |  |  | Ocean-going deepwater (freezer) trawlers | Demersal vessels |
|  |  |  | Ocean-going deepwater crustaceans freezer trawlers | Other crustaceans |
|  |  |  | Pole-and-line tuna vessels | Tuna liners |
|  |  |  | Freezer tuna seiners | Tuna seiners |
|  |  |  | Surface longliners | Tuna liners |
|  | July 2002 to June 2006 | [162] | Fish and cephalopods demersal trawlers | Demersal vessels |
|  |  |  | Ocean-going deepwater trawlers and bottom longliners | Demersal vessels |
|  |  |  | Ocean-going deepwater crustaceans freezer trawlers | Other crustaceans |
|  |  |  | Pole-and-line tuna vessels | Tuna liners |
|  |  |  | Freezer tuna seiners | Tuna seiners |
|  |  |  | Surface longliners | Tuna liners |
| Seychelles | January 1984 to December 1986 | [163,164] | Ocean-going freezer tuna boats | Tuna seiners |
|  | January 1987 to December 1990 | [165,166] | Ocean-going tuna vessels | Tuna seiners |
|  | January 1990 to December 1995 | [167,168] | Ocean-going tuna seiners | Tuna seiners |
|  | January 1996 to December 2012 | [169-173] | Ocean-going tuna seiners | Tuna seiners |
|  |  |  | Surface longliners | Tuna liners |
| Solomon Islands | October 2006 to September 2009 | [174] | Purse seine vessels | Tuna seiners |
|  |  |  | Surface longliners | Tuna liners |
|  | October 2009 to September 2012 | [175] | Purse seiners | Tuna seiners |
| ^a^ The list of full references is provided in **Supporting References S1**.  ^b^ For the 1983-1986 period, various additional fees for the industry could not be computed due to a lack of information. However, we believe they were marginal compared to the overall fee paid by the industry.  ^c^ Additional fees for the industry could not be computed due to the absence of a quotas in the agreement. | | | | |
|  | | | | |
