# Supplementary material for: European Union’s Public Fishing Access Agreements in Developing Countries
Source: PLoS One. 2013 Nov 27;8(11):e79899. doi: 10.1371/journal.pone.0079899 (PMC3842348; doi:10.1371/journal.pone.0079899)
Supplement: Table S2 — Correspondence between the number of vessels and GRT capacities for non-tuna vessels. (DOCX) [file pone.0079899.s005.docx]

| **Table S2**. Correspondence between the number of vessels and GRT capacities for non-tuna vessels. | | | |
| --- | --- | --- | --- |
| **Country** | **Period** | **Type of gear** | **Correspondence used** |
| Angola | 1996-2004 | Pelagic species | Mauritania [98] |
| Cape Verde | 1991-1994 | Cephalopods | Mauritania [95] |
| Côte d’Ivoire | 1997-2000 | Demersal trawlers | Angola [7] |
| Madagascar | 2007-2012 | Bottom longliners, fixed gillnets | Cape Verde [18] ^b^ |
| Mozambique | 1990-1991 | Demersal trawlers | Angola [4] ^b^ |
|  | 2003-2006 | Shrimp | Angola [10] ^b^ |
| ^a^ The list of full references is provided in **Supporting References S1**.  ^b^ In these cases, no correspondence in the same area was available. | | | |
