# Supplementary material for: European Union’s Public Fishing Access Agreements in Developing Countries
Source: PLoS One. 2013 Nov 27;8(11):e79899. doi: 10.1371/journal.pone.0079899 (PMC3842348; doi:10.1371/journal.pone.0079899)
Supplement: Table S3 — Assumptions made to fill the gaps in the CPI time-series. (DOCX) [file pone.0079899.s006.docx]

| **Table S3**. Assumptions made to fill the gaps in the CPI time-series. | | | |
| --- | --- | --- | --- |
| **Time-series** | **Country** | **Period** | **Comment** |
| CPI | Angola  Comoros  Equatorial Guinea  Guinea  Guinea-Bissau  Sao Tome and Principe  Kiribati  Micronesia | 1987-1989  1988-1999  1984  1983  1980-1986  1985-1985  2003-2012  2007-2012 | Next five years’ average annual growth rate applied backward ^a^  -  -  -  -  -  Pacific Small States Developing Islands values applied  - |
| ^a^ Complete CPI time-series have a sigmoid behavior over the period considered. Thus we assumed that using the average growth rates would provide us with a reasonable way to estimate missing values of incomplete time-series. In the cases of Comoros and Guinea, our CPI values are therefore likely over-estimated (i.e., under-estimates the effect of earlier years’ inflation on the 2012 EUR), as the time-series was not yet showing any inflexion point for the available years. | | | |
