# Supplementary material for: European Union’s Public Fishing Access Agreements in Developing Countries
Source: PLoS One. 2013 Nov 27;8(11):e79899. doi: 10.1371/journal.pone.0079899 (PMC3842348; doi:10.1371/journal.pone.0079899)
Supplement: References S1 — References used in Table S1 and Table S2. (DOCX) [file pone.0079899.s008.docx]

**Supporting References S1. References used in Table S1 and Table S2.**

1. European Union (1987) Protocol establishing the fishing rights and financial contribution provided for in the Agreement between the European Economic Community and the Government of the People's Republic of Angola on fishing off Angola. Official Journal L 341: 13-13.

2. European Union (1987) Agreement between the European Economic Community and the Government of the People's Republic of Angola on fishing off Angola. Official Journal L 341: 2-12.

3. European Union (1989) Protocol defining, for the period 3 May 1989 to 2 May 1990, the fishing opportunities and financial compensation provided for in the Agreement between the European Economic Community and the People's Republic of Angola on fishing off Angola. Official Journal L 341: 9-18.

4. European Union (1990) Protocol defining, for the period 3 May 1990 to 2 May 1992, the fishing opportunities and financial compensation provided for in the Agreement between the European Economic Community and the Government of the People's Republic of Angola on fishing off Angola. Official Journal L 319: 34-44.

5. European Union (1993) Protocol defining, for the period from 3 May 1992 to 2 May 1994, the fishing possibilities and financial compensation provided for in the Agreement between the European Community and the Government of the People' s Republic of Angola on fishing off Angola. Official Journal L 64: 4-14.

6. European Union (1994) Protocol defining, for the period from 3 May 1994 to 2 May 1996, the fishing possibilities and financial compensation provided for in the Agreement between the European Economic Community and the Government of the People's Republic of Angola on fishing off Angola. Official Journal L 324: 2-15.

7. European Union (1997) Protocol defining, for the period from 3 May 1996 to 2 May 1999, the fishing opportunities and financial compensation provided for in the Agreement between the European Economic Community and the Government of the People's Republic of Angola on fishing off Angola. Official Journal L 46: 57-75.

8. European Union (2000) Protocol Defining, for the period from 3 May 1999 to 2 May 2000, the fishing opportunities and financial compensation provided for in the Agreement between the European Community and the Government of the People's Republic of Angola on fishing off Angola. Official Journal L 17: 3-21.

9. European Union (2001) Protocol setting out for the period from 3 May 2000 to 2 May 2002 the fishing opportunities and financial contribution provided for in the Agreement between the European Economic Community and the Government of the People's Republic of Angola on fishing off Angola. Official Journal L 66: 3-21.

10. European Union (2002) Protocol setting out, for the period from 3 August 2002 to 2 August 2004, the fishing opportunities and the financial contribution provided for by the Agreement between the European Economic Community and the Government of the Republic of Angola on fishing off Angola. Official Journal L 351: 92-111.

11. European Union (2002) Council Regulation (EC) No 2057/2002 of 11 November 2002 on the conclusion of the Agreement in the form of an Exchange of Letters on the extension of the Protocol setting out the fishing rights and the financial contribution provided for by the Agreement between the European Economic Community and the Government of the Republic of Angola on fishing off Angola for the period from 3 May to 2 August 2002. Official Journal L 317: 12-13.

12. European Union (1990) Protocol establishing the fishing rights and financial compensation provided for in the Agreement between the European Economic Community and the Republic of Cap Verde on fishing off the coast of Cape Verde Official Journal L 212: 13-13.

13. European Union (1995) Protocol establishing the fishing rights and financial compensation provided for in the agreement between the European Economic Community and the Republic of Cape Verde on fishing off the coast of Cape Verde. Official Journal L 199: 10-18.

14. European Union (1990) Agreement between the European Economic Community and the Republic of Cape Verde on fishing off the coast of Cape Verde. Official Journal L 212: 3-12.

15. European Union (1998) Protocol establishing the fishing rights and financial compensation provided for in the Agreement between the European Economic Community and the Republic of Cape Verde on fishing off the coast of Cape Verde. Official Journal L 21: 20-27.

16. European Union (2002) Protocol setting out the fishing opportunities and financial contribution provided for in the Agreement between the European Economic Community and the Republic of Cape Verde on fishing off the coast of Cape Verde for the period from 1 July 2001 to 30 June 2004. Official Journal L 47: 25-33.

17. European Union (2004) Council Regulation (EC) No 1927/2004 of 21 October 2004 on the conclusion of the Agreement in the form of an Exchange of Letters concerning the extension of the Protocol setting out the fishing opportunities and financial contribution provided for in the Agreement between the European Economic Community and the Republic of Cape Verde on fishing off the coast of Cape Verde for the period 1 July 2004 to 30 June 2005. Official Journal L 332: 1-2.

18. European Union (2006) Protocol setting out the fishing opportunities and financial contribution provided for in the Fisheries Partnership Agreement between the European Community and the Republic of Cape Verde on fishing off the Coast of Cape Verde for the period from 1 September 2006 to 31 August 2011. Official Journal L 414: 8-25.

19. European Union (2011) Protocol agreed between the European Union and the Republic of Cape Verde setting out the fishing opportunities and the financial contribution provided for in the Fisheries Partnership Agreement between the two parties currently in force. Official Journal L 181: 2-19.

20. European Union (1988) Protocol setting out the fishing opportunities and financial compensation provided for under the Agreement between the European Economic Community and the Islamic Federal Republic of the Comoros on fishing off Comoros. Official Journal L 137: 24-24.

21. European Union (1992) Protocol setting out the fishing opportunities and financial contribution provided for in the Agreement between the European Economic Community and the Islamic Federal Republic of the Comoros on fishing off the Comoros for the period 20 July 1991 to 19 July 1994. Official Journal L 288: 2-3.

22. European Union (1995) Protocol setting out the fishing opportunities and financial consideration provided for in the Agreement between the European Economic Community and the Islamic Federal Republic of the Comoros on fishing off Comoros for the period 20 July 1994 to 19 July 1997. Official Journal L 180: 9-13.

23. European Union (1988) Agreement between the European Economic Community and the Islamic Federal Republic of the Comoros on fishing off Comoros. Official Journal L 137: 19-23.

24. European Union (1998) Protocol setting out, for the period 28 February 1998 to 27 February 2001, the fishing opportunities and financial contribution provided for in the Agreement between the European Economic Community and the Islamic Federal Republic of the Comoros on fishing off the Comoros. Official Journal L 217: 30-34.

25. European Union (2001) Protocol setting out the fishing opportunities and financial contribution provided for in the Agreement between the European Economic Community and the Islamic Federal Republic of the Comoros on fishing off the Comoros for the period from 28 February 2001 to 27 February 2004. Official Journal L 193: 19-23.

26. European Union (2005) Protocol setting out the fishing opportunities and financial contribution provided for in the Agreement between the European Economic Community and the Islamic Federal Republic of the Comoros on fishing off the Comoros for the period from 1 January 2005 to 31 December 2010. Official Journal L 252: 11-26.

27. European Union (2010) Protocol setting out the fishing opportunities and financial contribution provided for in the Partnership Agreement in the fisheries sector between the European Community and the Union of the Comoros. Official Journal L 335: 3-18.

28. European Union (1990) Protocol establishing the fishing rights and financial compensation provided for in the Agreement between the European Economic Community and the Republic of Côte d'Ivoire on fishing off the coast of Côte d'Ivoire. Official Journal L 379: 14-14.

29. European Union (1995) Protocol establishing the fishing rights and financial compensation provided for in the Agreement between the European Economic Community and the Republic of Côte d'Ivoire on fishing off the coast of Côte d'Ivoire. Official Journal L 180: 15-26.

30. European Union (2001) Protocol setting out, for the period 1 July 2000 to 30 June 2003, the fishing opportunities and financial contribution provided for in the Agreement between the European Economic Community and the Republic of Côte d'Ivoire on fishing off the coast of Côte d'Ivoire. Official Journal L 102: 3-14.

31. European Union (1998) Protocol establishing the fishing rights and financial contribution provided for in the Agreement between the European Economic Community and the Republic of Côte d'Ivoire on fishing off the coast of Côte d'Ivoire. Official Journal L 25: 85-95.

32. European Union (2004) Council Regulation (EC) No 154/2004 of 26 January 2004 on the conclusion of an Agreement in the form of an Exchange of Letters extending for the period 1 July 2003 to 30 June 2004 the validity of the Protocol setting fishing opportunities and a financial contribution as provided for in the Agreement between the European Economic Community and the Republic of Côte d'Ivoire on fishing off the coast of Côte d'Ivoire. Official Journal L 27: 1-2.

33. European Union (2005) Protocol setting out, for the period from 1 July 2004 to 30 June 2007, the fishing opportunities and financial contribution provided for in the agreement between the European Economic Community and the Republic of Côte d'Ivoire on fishing off the coast of Côte d'Ivoire. Official Journal L 76: 4-15.

34. European Union (1990) Agreement between the European Economic Community and the Republic of Côte d'Ivoire on fishing off the coast of Côte d'Ivoire. Official Journal L 379: 3-13.

35. European Union (2008) Protocol setting out the fishing opportunities and the financial contribution provided for by the Agreement between the European Community and the Republic of Côte d’Ivoire on fishing off the coast of Côte d’Ivoire for the period from 1 July 2007 to 30 June 2013. Official Journal L 48: 46-63.

36. European Union (1984) Protocol between the European Economic Community and the Government of the Republic of Equatorial Guinea. Official Journal L 188: 6-6.

37. European Union (1984) Agreement between the European Economic Community and the Government of the Republic of Equatorial Guinea on fishing off the coast of Equatorial Guinea. Official Journal L 188: 2-5.

38. European Union (1987) Protocol establishing fishing rights and financial compensation for the period from 27 June 1986 to 26 June 1989. Official Journal L 29: 8-8.

39. European Union (1987) Agreement amending the Agreement between the European Economic Community and the Government of the Republic of Equatorial Guinea on fishing off the coast of Equatorial Guinea, signed at Malabo on 15 June 1984. Official Journal L 29: 3-7.

40. European Union (1989) Protocol establishing, for the period from 27 June 1989 to 26 June 1992, the fishing rights and financial compensation provided for in the Agreement between the European Economic Community and the Government of the Republic of Equatorial Guinea on fishing off the coast of Equatorial Guinea. Official Journal L 299.

41. European Union (1995) Protocol establishing for the period from 1 July 1994 to 30 June 1997 the fishing rights and financial compensation provided for in the Agreement between the European Economic Community and the Government of the Republic of Equatorial Guinea on fishing off the coast of Equatorial Guinea. Official Journal L 180: 2-7.

42. European Union (1998) Protocol establishing, for the period from 1 July 1997 to 30 June 2000, the fishing rights and financial compensation provided for in the Agreement between the European Economic Community and the Government of the Republic of Equatorial Guinea on fishing off the coast of Equatorial Guinea. Official Journal L 11: 33-38.

43. European Union (2000) Protocol establishing, for the period 1 July 2000 to 30 June 2001, the fishing rights and financial compensation provided for in the Agreement between the European Economic Community and the Government of the Republic of Equatorial Guinea on fishing off the coast of Equatorial Guinea. Official Journal L 329: 41-45.

44. European Union (1998) Protocol setting out the fishing opportunities and financial compensation provided for in the Agreement between the European Community and the Gabonese Republic on fishing off the coast of Gabon. Official Journal L 308: 6-11.

45. European Union (2002) Protocol setting out the fishing opportunities and the financial contribution provided for by the Agreement between the European Community and the Gabonese Republic on fishing off the coast of Gabon for the period 3 December 2001 to 2 December 2005. Official Journal L 73: 19-29.

46. European Union (2006) Protocol setting out the fishing opportunities and financial contribution provided for in the Agreement between the European Community and the Gabonese Republic on fishing off the coast of Gabon for the period from 3 December 2005 to 2 December 2011. Official Journal L 319: 18-36.

47. European Union (1987) Protocol between the European Economic Community and the Government of the Republic of the Gambia. Official Journal L 146: 10-11.

48. European Union (1987) Agreement between the European Economic Community and the Government of the Republic of the Gambia on fishing off the Gambia. Official Journal L 146: 3-9.

49. European Union (1990) Protocol establishing for the period from 1 July 1990 to 30 June 1993 the fishing rights and financial compensation provided for in the Agreement between the European Economic Community and the Republic of the Gambia on fishing off the Gambia. Official Journal L 379: 17-23.

50. European Union (1994) Protocol establishing the fishing rights and financial compensation provided for in the Agreement between the European Economic Community and the Republic of The Gambia on fishing off the coast of The Gambia for the period 1 July 1993 to 30 June 1996. Official Journal L 79: 2-10.

51. European Union (1980) Agreement between the Government of the Republic of Guinea Bissau and the European Economic Community on fishing off the coast of Guinea Bissau. Official Journal L 226: 34-41.

52. European Union (1980) Protocol between the European Economic Community and the Government of the Republic of Guinea Bissau. Official Journal L 226: 42-42.

53. European Union (1982) Council Decision of 26 April 1982 on the conclusion of the Agreement in the form of an exchange of letters concerning an interim extension of the Protocol annexed to the Agreement between the Government of the Republic of Guinea Bissau and the European Economic Community on fishing off the coast of Guinea Bissau. Official Journal L 126: 16-16.

54. European Union (1982) Council Decision of 28 July 1982 concerning the conclusion of the Agreement in the form of an exchange of letters concerning a second interim extension of the Protocol annexed to the Agreement between the Government of Guinea Bissau and the European Economic Community on fishing off the coast of Guinea Bissau. Official Journal L 247: 33-33.

55. European Union (1983) Protocol between the European Economic Community and the Government of the Republic of Guinea Bissau. Official Journal L 84: 2-8.

56. European Union (1987) Protocol establishing rights and financial compensation for the period from 16 June 1986 to 15 June 1989. Official Journal L 113: 10-10.

57. European Union (1987) Agreement amending for the second time the Agreement between the European Economic Community and the Government of the Republic of Guinea-Bissau on fishing off the coast of Guinea-Bissau. Official Journal L 113: 3-9.

58. European Union (1990) Protocol establishing for the period 16 June 1989 to 15 June 1991 the fishing rights and financial compensation provided for in the Agreement between the European Economic Community and the Government of the Republic of Guinea-Bissau on fishing off the coast of Guinea-Bissau. Official Journal L 125: 3-16.

59. European Union (1991) Protocol establishing for the period 16 June 1991 to 15 June 1993 the fishing rights and financial compensation provided for in the Agreement between the European Economic Community and the Government of the Republic of Guinea-Bissau on fishing off the coast of Guinea-Bissau. Official Journal L 309: 8-18.

60. European Union (1994) Protocol establishing for the period 16 June 1993 to 15 June 1995 the fishing rights and financial compensation provided for in the Agreement between the European Economic Community and the Government of the Republic of Guinea-Bissau on fishing off the coast of Guinea-Bissau. Official Journal L 60: 2-13.

61. European Union (1996) Protocol establishing the fishing possibilities and the financial compensation provided for in the agreement between the European Economic Community and the Government of the Republic of Guinea- Bissau on fishing off the coast of Guinea-Bissau for the period 16 June 1995 to 15 June 1997 - Annex: Conditions governing fishing by community vessels in Guinea Bissau's fishing zone. Official Journal L 85: 3-16.

62. European Union (1997) Protocol establishing the fishing possibilities and the financial compensation provided for in the agreement between the European Economic Community and the Government of the Republic of Guinea-Bissau on fishing off the coast of Guinea-Bissau for the period 16 June 1997 to 15 June 2001. Official Journal L 342: 4-17.

63. European Union (2002) Protocol establishing the fishing opportunities and the compensation provided for in the Agreement between the European Economic Community and the Government of the Republic of Guinea-Bissau on fishing off the coast of Guinea-Bissau for the period 16 June 2001 to 15 June 2006. Official Journal L 19: 35-46.

64. European Union (2006) Agreement in the form of an Exchange of Letters concerning the extension of the Protocol establishing the fishing opportunities and the financial contribution provided for in the Agreement between the European Economic Community and the Government of the Republic of Guinea-Bissau on fishing off the coast of Guinea-Bissau for the period 16 June 2006 to 15 June 2007. Official Journal L 2006: 9-10.

65. European Union (2007) Protocol setting out the fishing opportunities and financial contribution provided for in the Fisheries Partnership Agreement between the European Community and the Republic of Guinea-Bissau for the period 16 June 2007 to 15 June 2011. Official Journal L 342: 10-37.

66. European Union (1983) Protocol defining the fishing rights and financial compensation provided for in the Agreement between the European Economic Community and the Revolutionary People's Republic of Guinea. Official Journal L 111: 17-17.

67. European Union (1986) Council Decision of 18 March 1986 on the conclusion of an Agreement in the form of an exchange of letters on the interim extension of the Protocol annexed to the Agreement between the European Economic Community and the Government of the Republic of Guinea on fishing off the Guinean coast for a six-month period as from 8 February 1986. Official Journal L 80: 52-52.

68. European Union (1983) Agreement between the European Economic Community and the Government of the Revolutionary People's Republic of Guinea on fishing off the Guinean coast. Official Journal L 111: 2-16.

69. European Union (1987) Agreement between the European Economic Community and the Government of the Republic of Guinea amending the Agreement between the European Economic Community and the Government of the Revolutionary People's Republic of Guinea on fishing off the coast of Guinea, signed at Conakry on 7 February 1983. Official Journal L 29: 10-15.

70. European Union (1987) Protocol establishing fishing rights and financial compensation for the period from 8 August 1986 to 7 August 1989. Official Journal L 29: 16-16.

71. European Union (1990) Protocol establishing, for the period 1 January 1990 to 31 December 1991, the fishing rights and financial compensation provided for in the Agreement between the European Economic Community and the Government of the Republic of Guinea on fishing off the Guinean coast. Official Journal L 212: 16-27.

72. European Union (1992) Protocol establishing, for the period from 1 January 1992 to 31 December 1993, the fishing rights and financial compensation provided for in the Agreement between the European Economic Community and the Government of the Republic of Guinea on fishing off the Guinean coast. Official Journal L 379: 2-14.

73. European Union (1995) Protocol establishing, for the period from 1 January 1994 to 31 December 1995, the fishing rights and financial compensation provided for in the Agreement between the European Economic Community and the Government of the Republic of Guinea on fishing off the Guinean coast. Official Journal L 278: 2-13.

74. European Union (1996) Protocol establishing, for the period from 1 January 1996 to 31 December 1997, the fishing rights and financial compensation provided for in the Agreement between the European Economic Community and the Government of the Republic of Guinea on fishing off the Guinea coast. Official Journal L 157: 3-16.

75. European Union (1998) Protocol establishing the fishing possibilities and the financial compensation provided for in the Agreement between the European Economic Community and the Government of the Revolutionary People's Republic of Guinea on fishing off the Guinean coast for the period 1 January 1998 to 31 December 1999. Official Journal L 196: 32-45.

76. European Union (2000) Protocol setting out the fishing rights and financial contribution provided for in the Agreement between the European Economic Community and the Government of the Republic of Guinea on fishing off the coast of Guinea for the period 1 January 2000 to 31 December 2001. Official Journal L 250: 32-45.

77. European Union (2002) Council Regulation (EC) No 924/2002 of 30 May 2002 on the conclusion of the Agreement in the form of an Exchange of Letters concerning the extension of the 2000-2001 Protocol setting out the fishing opportunities and financial contribution provided for in the Agreement between the European Economic Community and the Government of the Revolutionary People's Republic of Guinea on fishing off the Guinean coast for the period 1 January 2002 to 31 December 2002. Official Journal L 144: 3-4.

78. European Union (2004) Protocol defining for the period 1 January 2004 to 31 December 2008 the fishing opportunities and financial contribution provided for in the Agreement between the European Economic Community and the Republic of Guinea on fishing off the coast of Guinea. Official Journal L 99: 12-27.

79. European Union (2009) Protocol setting out the fishing opportunities and financial contribution provided for in the Agreement between the European Community and the Republic of Guinea on fishing off the coast of Guinea for the period from 1 January 2009 to 31 December 2012. Official Journal L 156: 35-55.

80. European Union (2003) Protocol setting out the fishing possibilities and the financial contribution provided for in the Agreement between the European Community and the Republic of Kiribati on fishing within the Kiribati fishing zone. Official Journal L 126: 5-19.

81. European Union (2007) Protocol setting out the fishing opportunities and financial contribution provided for in the Fisheries Partnership Agreement between the European Community and the Republic of Kiribati for the period from 16 September 2006 to 15 September 2012. Official Journal L 205: 8-34.

82. European Union (2012) Protocol setting out the fishing opportunities and financial contribution provided for in the Fisheries Partnership Agreement between the European Community, on the one hand, and the Republic of Kiribati, on the other. Official Journal L 300: 3-33.

83. European Union (1986) Protocol 1 between the European Economic Community and the Government of the Democratic Republic of Madagascar on tuna fishing. Official Journal L 73: 31-31.

84. European Union (1986) Protocol 2 between the European Economic Community and the Government of the Democratic Republic of Madagascar on types of fishing other than those covered by Protocol 1. Official Journal L 73: 32-32.

85. European Union (1986) Agreement between the European Economic Community and the Government of the Democratic Republic of Madagascar on fishing off Madagascar. Official Journal L 73: 26-30.

86. European Union (1989) Protocol defining, for the period 21 May 1989 to 20 May 1992, the fishing opportunities and the financial contribution provided for by the agreement between the European Economic Community and the government of the Democratic Republic of Madagascar on fishing off Madagascar. Official Journal L 239: 3-8.

87. European Union (1993) Protocol defining for the period 21 May 1992 to 20 May 1995 the fishing opportunities and the financial contribution provided for by the agreement between the European Community and the Government of the Democratic Republic of Madagascar on fishing off Madagascar. Official Journal L 106: 2-6.

88. European Union (1996) Protocol defining, for the period 21 May 1995 to 20 May 1998, the fishing opportunities and the financial contribution provided for by the Agreement between the European Economic Community and the Government of the Republic of Madagascar on fishing off Madagascar. Official Journal L 75: 2-7.

89. European Union (1998) Protocol setting out the fishing opportunities and financial compensation provided for in the Agreement between the European Economic Community and the Republic of Madagascar on fishing off Madagascar for the period from 21 May 1998 to 20 May 2001. Official Journal L 295: 34-39.

90. European Union (2001) Protocol setting out the fishing opportunities and financial contribution provided for in the agreement between the European Economic Community and the Democratic Republic of Madagascar on fishing off Madagascar for the period from 21 May 2001 to 20 May 2004. Official Journal L 296: 10-18.

91. European Union (2005) Protocol defining for the period 1 January 2004 to 31 December 2006 the Tuna fishing opportunities and the financial contribution provided for in the agreement between the European Economic Community and the Democratic Republic of Madagascar on fishing off Madagascar. Official Journal L 94: 5-44.

92. European Union (2007) Protocol setting out the fishing opportunities and financial contribution provided for in the Agreement between the European Community and the Republic of Madagascar on fishing off the coast of Madagascar for the period from 1 January 2007 to 31 December 2012. Official Journal L 331: 11-30.

93. European Union (1987) Protocol setting out fishing opportunities and financial compensation for the period 1 July 1987 to 30 June 1990. Official Journal L 302: 34-35.

94. European Union (1987) Agreement between the European Economic Community and the Islamic Republic of Mauritania on fishing off the coast of Mauritania. Official Journal L 302: 26-33.

95. European Union (1990) Protocol setting out the fishing opportunities and financial contribution provided for in the Agreement between the European Economic Community and the Islamic Republic of Mauritania on fishing off the coast of Mauritania for the period 1 August 1990 to 31 July 1993. Official Journal L 334: 12-22.

96. European Union (1993) Protocol setting out the fishing opportunities and financial contribution provided for in the Agreement between the European Community and the Islamic Republic of Mauritania on fishing off the coast of Mauritania for the period 1 August 1993 to 31 July 1996. Official Journal L 290: 20-31.

97. European Union (1996) Supplement to the Protocol setting out the fishing opportunities and financial contribution provided for in the Agreement between the European Economic Community and the Islamic Republic of Mauritania on fishing off the coast of Mauritania, for the period 15 November 1995 to 31 July 1996. Official Journal L 76: 27-28.

98. European Union (1996) Protocol setting out fishing opportunities and the financial compensation and financial contributions for the period 1 August 1996 to 31 July 2001. Official Journal L 334: 24-54.

99. European Union (2001) Protocol setting out the fishing opportunities and financial compensation provided for in the Agreement on cooperation in the sea fisheries sector between the European Community and the Islamic Republic of Mauritania for the period 1 August 2001 to 31 July 2006. Official Journal L 341: 128-159.

100. European Union (2006) Protocol setting out the fishing opportunities and financial contribution provided for in the Fisheries Partnership Agreement between the European Community and the Islamic Republic of Mauritania. Official Journal L 343: 9-60.

101. European Union (2008) Protocol setting out the fishing opportunities and financial contribution provided for in the Fisheries Partnership Agreement between the European Community and the Islamic Republic of Mauritania for the period 1 August 2008 to 31 July 2012. Official Journal L 203: 4-59.

102. European Union (2012) Protocol setting out the fishing opportunities and financial contribution provided for in the fisheries partnership Agreement between the European Union and the Islamic Republic of Mauritania for a period of two years. Official Journal L 361: 44-84.

103. European Union (1989) Protocol No 1 on the fishing opportunities accorded by Mauritius and the financial contribution accorded by the Community. Official Journal L 159: 7-7.

104. European Union (1989) Protocol No 2 on experimental fishing for crustacea. Official Journal L 159: 8-8.

105. European Union (1989) Agreement between the European Economic Community and the Government of Mauritius on fishing in Mauritian waters. Official Journal L 159: 2-6.

106. European Union (1994) Protocol defining, for the period 1 December 1993 to 30 November 1996, the fishing opportunities and the financial contribution provided for by the Agreement between the European Community and the Government of Mauritius on fishing in the waters of Mauritius - Annex: Conditions for the pursuit of fishing activities by Community vessels in the waters of Mauritius. Official Journal L 187: 4-7.

107. European Union (1997) Protocol fixing, for the period 1 December 1996 to 30 November 1999, the fishing opportunities and the financial consideration provided for in the Agreement between the European Economic Community and the Government of Mauritius on fishing in Mauritian waters. Official Journal L 163: 29-33.

108. European Union (2000) Protocol defining, for the period 3 December 1999 to 2 December 2002, the fishing opportunities and the financial contribution provided for by the Agreement between the European Community and the Government of Mauritius on fishing in the waters of Mauritius. Official Journal L 180: 30-38.

109. European Union (2003) Council Regulation (EC) No 1869/2003 of 20 October 2003 on the conclusion of the Agreement in the form of an Exchange of Letters concerning the extension of the Protocol setting out the fishing opportunities and financial contribution provided for in the Agreement between the European Community and the Government of Mauritius on fishing in Mauritian waters for the period 3 December 2002 to 2 December 2003. Official Journal L 275: 1-2.

110. European Union (2004) Protocol defining, for the period 3 December 2003 to 2 December 2007, the fishing opportunities and the financial compensation provided for by the Agreement between the European Economic Community and the Government of Mauritius on fishing in Mauritian waters. Official Journal L 348: 3-13.

111. European Union (2006) Protocol setting out the fishing opportunities and financial contribution provided for in the Partnership Agreement between the European Community and the Federated States of Micronesia on fishing in the Federated States of Micronesia. Official Journal L 151: 8-30.

112. European Union (2011) Protocol setting out the fishing opportunities and financial contribution provided for in the Fisheries Partnership Agreement between the European Community and the Federated States of Micronesia on fishing in the Federated States of Micronesia. Official Journal L 52: 66-67.

113. European Union (1988) Agreement on relations in the sea fisheries sector between the European Economic Community and the Kingdom of Morocco. Official Journal L 99: 3-13.

114. European Union (1988) Protocol 1 setting out fishing opportunities accorded by Morocco and the compensation accorded by the Community for the period from 1 March 1988 to 29 February 1992. Official Journal L 99: 14-16.

115. European Union (1988) Protocol 2 on experimental fishing. Official Journal L 99: 17-17.

116. European Union (1992) Agreement in the form of an exchange of letters concerning the interim extension for the period from March 1 1992 to 30 April 1992 of the agreements on relations in the sea-fisheries sector between the European Economic Community and the Kingdom of Morocco and of the Protocol annexed thereto. Official Journal L 91: 28-29.

117. European Union (1992) Protocol setting out fishing opportunities and the financial compensation and financial contributions accorded by the Community. Official Journal L 407: 15-28.

118. European Union (1992) Agreement on relations in the sea fisheries sector between the European Economic Community and the Kingdom of Morocco. Official Journal L 407: 3-14.

119. European Union (1995) Protocole fixant les possibilités de pêche et les montants de la compensation financière et des appuis financiers. Official Journal L 306: 32-43.

120. European Union (1995) Council Decision of 7 December 1995 on the conclusion of an Agreement in the form of an exchange of letters concerning the provisional application of the Agreement on cooperation in the sea fisheries sector between the European Community and the Kingdom of Morocco initialled in Brussels on 13 November 1995. Official Journal L 306: 1-4.

121. European Union (2006) Protocol setting out the fishing opportunities and financial contribution provided for in the Fisheries Partnership Agreement between the European Community and the Kingdom of Morocco. Official Journal L 141: 9-37.

122. European Union (2011) Protocol between the European Union and the Kingdom of Morocco setting out the fishing opportunities and financial compensation provided for in the Fisheries Partnership Agreement between the European Community and the Kingdom of Morocco. Official Journal L 202: 3-30.

123. European Union (1987) Protocol establishing the fishing rights and contributions provided for in the Agreement between the European Economic Community and the Government of the People's Republic of Mozambique on fisheries relations. Official Journal L 201: 14-14.

124. European Union (1987) Agreement between the European Economic Community and the Government of the People's Republic of Mozambique on fisheries relations. Official Journal L 201: 2-13.

125. European Union (1990) Protocol establishing, for the period 1 January 1990 to 31 December 1991, the fishing opportunities and the financial contribution provided for in the Agreement between the European Economic Community and the Government of the People's Republic of Mozambique on fisheries relations. Official Journal L 140: 4-5.

126. European Union (1990) Corrigendum to Council Regulation (EEC) No 1454/90 of 28 May 1990 relating to the conclusion of the Protocol establishing, for the period 1 January 1990 to 31 December 1991, the fishing opportunities and the financial contribution provided for in the Agreement between the European Economic Community and the Government of the People's Republic of Mozambique on fisheries relations (OJ No L 140 of 1. 6. 1990). Official Journal L 35: 33-33.

127. European Union (1993) Protocol establishing, for the period from 1 January 1992 to 30 September 1993 the fishing opportunities and financial contribution provided for in the Agreement between the European Economic Community and the People's Republic of Mozambique on fisheries relations. Official Journal L 64: 2-2.

128. European Union (2003) Protocol setting out the fishing opportunities and financial contribution provided for in the Fisheries Agreement between the European Community and the Republic of Mozambique. Official Journal L 345: 48-63.

129. European Union (2007) Protocol Partnership Agreement between the European Community and the Republic of Mozambique on fishing off the coast of Mozambique for the period from 1 January 2007 to 31 December 2011. Official Journal L 331: 38-54.

130. European Union (2012) Protocol setting out the fishing opportunities and the financial contribution provided for by the Fisheries Partnership Agreement between the European Community and the Republic of Mozambique. Official Journal L 46: 4-29.

131. European Union (1984) Protocol between the European Economic Community and the Government of the Democratic Republic of São Tomé and Principe. Official Journal L 54: 5-5.

132. European Union (1986) Council Decision of 3 December 1986 on the conclusion of an Agreement in the form of an Exchange of Letters concerning an extension of the Protocol annexed to the Agreement between the European Economic Community and the Government of the Democratic Republic of Sao Tomé and Principe on fishing off the coast of Sao Tomé and Principe. Official Journal L 344: 28-28.

133. European Union (1984) Agreement between the European Economic Community and the Government of the Democratic Republic of São Tomé and Principe on fishing off São Tomé and Principe. Official Journal L 54: 2-4.

134. European Union (1987) Protocol establishing fishing authorizations and financial compensation for the period from 1 June 1987 to 31 May 1990. Official Journal L 300: 37-37.

135. European Union (1990) Protocol establishing fishing rights and financial compensation for the period 1 June 1990 to 31 May 1993. Official Journal L 334: 4-8.

136. European Union (1987) Agreement amending the Agreement between the European Economic Community and the Government of the Democratic Republic of São Tomé and Principe on fishing off São Tomé and Principe signed at Brussels on 1 February 1984. Official Journal L 300: 34-36.

137. European Union (1993) Protocol establishing fishing rights and financial compensation provided for in the Agreement between the European Economic Community and the Government of the Democratic Republic of São Tomé e Príncipe on fishing off São Tomé e Príncipe for the period 1 June 1993 to 31 May 1996. Official Journal L 292: 2-6.

138. European Union (1997) Protocol establishing the fishing opportunities and the financial contribution provided for in the Agreement between the European Community and the Government of the Democratic Republic of São Tomé e Príncipe on fishing off the coast of São Tomé e Príncipe for the period 1 June 1996 to 31 May 1999. Official Journal L 46: 76-81.

139. European Union (2000) Protocol setting out, for the period 1 June 1999 to 31 May 2002, the fishing opportunities and the financial contribution provided for by the Agreement between the European Economic Community and the Government of the Democratic Republic of São Tomé e Príncipe on fishing off the Coast of São Tomé e Príncipe. Official Journal L 54: 3-9.

140. European Union (2002) Protocol setting out, for the period from 1 June 2002 to 31 May 2005, the fishing opportunities and the financial contribution provided for by the Agreement between the European Economic Community and the Government of the Democratic Republic of São Tomé and Príncipe on fishing off the coast of São Tomé and Príncipe. Official Journal L 351: 14-23.

141. European Union (2006) Council Regulation (EC) No 1124/2006 of 11 July 2006 on the conclusion of the Agreement in the form of an exchange of letters extending the Protocol setting out, for the period 1 June 2005 to 31 May 2006 , the fishing opportunities and the financial contribution provided for by the Agreement between the European Economic Community and the Government of the Democratic Republic of São Tomé and Príncipe on fishing off the coast of São Tomé e Príncipe. Official Journal L 200: 1-2.

142. European Union (2007) Protocol setting out the fishing opportunities and the financial contribution provided for by the Agreement between the Democratic Republic of São Tomé and Príncipe and the European Community on fishing off the coast of São Tomé and Príncipe for the period from 1 June 2006 to 31 May 2010. Official Journal L 205: 40-58.

143. European Union (2011) Protocol setting out the fishing opportunities and financial contribution provided for in the Fisheries Partnership Agreement between the European Union and the Democratic Republic of São Tomé and Príncipe. Official Journal L 136: 5-23.

144. European Union (1980) Protocol between the European Economic Community and the Government of the Republic of Senegal. Official Journal L 226: 28-32.

145. European Union (1981) Council Decision of 27 July 1981 on the conclusion of the Agreement in the form of an exchange of letters concerning an interim extension of the Protocol annexed to the Agreement between the Government of the Republic of Senegal and the European Economic Community on fishing off the coast of Senegal. Official Journal L 220: 34-34.

146. European Union (1980) Agreement between the Government of the Republic of Senegal and the European Economic Community on fishing off the coast of Senegal. Official Journal L 226: 17-27.

147. European Union (1982) Agreement between the European Economic Community and the Government of the Republic of Senegal amending the Agreement on fishing off the coast of Senegal, signed on 15 June 1979. Official Journal L 234: 9-10.

148. European Union (1984) Council Decision of 31 January 1984 on the conclusion of an Agreement in the form of an exchange of letters concerning an interim extension of the Protocol annexed to the Agreement between the Government of the Republic of Senegal and the European Economic Community on fishing off the coast of Senegal for the period 16 November 1983 to 15 January 1984. Official Journal L 37.

149. European Union (1985) Protocol establishing the fishing rights and compensation provided for in the Agreement between the European Economic Community and the Government of the Republic of Senegal on fishing off the coast of Senegal for the period 16 January 1984 to 15 January 1986. Official Journal L 361: 88-88.

150. European Union (1986) Council Decision of 18 June 1986 on the conclusion of an Agreement in the form of an exchange of letters concerning an interim extension of the Protocol annexed to the Agreement between the Government of the Republic of Senegal and the European Economic Community on fishing off the coast of Senegal for the period 1 to 31 May 1986. Official Journal L 1968: 22-22.

151. European Union (1986) Council Decision of 24 February 1986 on the conclusion of an Agreement in the form of an exchange of letters concerning an interim extension of the Protocol annexed to the Agreement between the Government of the Republic of Senegal and the European Economic Community on fishing off the coast of Senegal for the period 16 January to 30 April 1986. Official Journal L 75: 28-28.

152. European Union (1985) Agreement between the European Economic Community and the Government of the Republic of Senegal amending, for the second time, the agreement on fishing off the coast of Senegal. Official Journal L 361: 87-87.

153. European Union (1987) Protocol establishing the fishing rights and financial compensation provided for in the Agreement between the European Economic Community and the Government of the Republic of Senegal on fishing off the coast of Senegal for the period from 1 October 1986 to 28 February 1988. Official Journal L 57: 3-5.

154. European Union (1988) Protocol setting out the fishing rights and financial compensation provided for in the Agreement between the Government of the Republic of Senegal and the European Economic Community on fishing off the coast of Senegal for the period from 29 February 1988 to 28 February 1990. Official Journal L 137: 3-17.

155. European Union (1990) Council Decision of 27 July 1990 on the conclusion of an Agreement in the form of an Exchange of Letters temporarily extending from 1 to 30 April 1990 the Protocol to the Agreement between the European Economic Community and the Government of the Republic of Senegal on fishing off the coast of Senegal. Official Journal L 208: 31-31.

156. European Union (1991) Protocol defining, for the period 1 May 1990 to 30 April 1992, the fishing rights and financial compensation provided for in the Agreement between the European Economic Community and the Government of the Republic of Senegal on fishing off the coast of Senegal. Official Journal L 53: 3-18.

157. European Union (1993) Protocol defining, for the period 2 October 1992 to 1 October 1994, the fishing rights and financial compensation provided for in the Agreement between the European Economic Community and the Government of the Republic of Senegal on fishing off the coast of Senegal. Official Journal L 212: 2-16.

158. European Union (1995) Protocol establishing the fishing possibilities and financial compensation provided for in the Agreement between the European Economic Community and the Government of the Republic of Senegal on fishing off the coast of Senegal for the period from 2 October 1994 to 1 October 1996. Official Journal L 193: 6-21.

159. European Union (1997) Council Decision of 24 July 1997 on the conclusion of an Agreement in the form of an exchange of letters concerning the interim extension of the Protocol to the Agreement between the European Economic Community and the Government of the Republic of Senegal on fishing off the coast of Senegal for the period from 2 October 1996 to 1 November 1996. Official Journal L 216: 80-80.

160. European Union (1998) Protocol establishing the fishing rights and financial compensation provided for in the Agreement between the European Economic Community and the Government of the Republic of Senegal on fishing off the coast of Senegal for the period from 1 May 1997 to 30 April 2001. Official Journal L 302: 4-22.

161. European Union (2001) Council Regulation (EC) No 2303/2001 of 15 November 2001 on the conclusion of two Agreements in the form of Exchanges of Letters concerning the extension of the Protocol establishing the fishing opportunities and financial compensation provided for in the Agreement between the European Economic Community and the Government of the Republic of Senegal on fishing off the coast of Senegal for the periods 1 May 2001 to 31 July 2001 and 1 August 2001 to 31 December 2001. Official Journal L 310: 6-7.

162. European Union (2002) Protocol setting out the fishing opportunities and the financial contribution provided for in the Agreement between the European Economic Community and the Government of the Republic of Senegal on fishing off the coast of Senegal for the period from 1 July 2002 to 30 June 2006. Official Journal L 349: 46-65.

163. European Union (1985) Protocol between the European Economic Community and the Government of the Republic of Seychelles. Official Journal L 149: 6-6.

164. European Union (1985) Agreement between the European Economic Community and the Government of the Republic of Seychelles on fishing off Seychelles. Official Journal L 149: 2-5.

165. European Union (1987) Protocol on the fishing rights and financial compensation provided for in the Agreement between the European Economic Community and the Republic of Seychelles on fishing off Seychelles. Official Journal L 160: 10-10.

166. European Union (1987) Agreement between the European Economic Community and the Republic of Seychelles on fishing off Seychelles. Official Journal L 160: 2-9.

167. European Union (1990) Protocol defining, for the period from 18 January 1990 to 17 January 1993, the fishing opportunities and the financial contribution provided for by the Agreement between the European Economic Community and the Republic of Seychelles on fishing off Seychelles. Official Journal L 306: 2-5.

168. European Union (1993) Protocol defining for the period 18 January 1993 to 17 January 1996 the fishing opportunities and the financial contribution provided for by the Agreement between the European Community and the Republic of Seychelles on fishing off Seychelles. Official Journal L 246: 7-10.

169. European Union (1996) Protocol, defining for the period from 18 January 1996 to 17 January 1999, the fishing opportunities and financial contribution provided for by the Agreement between the European Economic Community and the Republic of Seychelles on fishing off Seychelles. Official Journal L 157: 19-26.

170. European Union (1999) Protocol defining for the period 18 January 1999 to 17 January 2002 the fishing opportunities and the financial contribution provided for by the agreement between the European Economic Community and the Republic of Seychelles on fishing off Seychelles. Official Journal L 131: 53-60.

171. European Union (2002) Protocol defining, for the period 18 January 2002 to 17 January 2005, the fishing possibilities and the financial contribution provided for by the Agreement between the European Economic Community and the Republic of Seychelles on fishing off Seychelles. Official Journal L 134: 40-47.

172. European Union (2005) Protocol setting out, for the period from 18 January 2005 to 17 January 2011, the fishing opportunities and the financial contribution provided for by the agreement between the European Economic Community and the Republic of Seychelles on fishing off Seychelles. Official Journal L 348: 4-25.

173. European Union (2010) Protocol setting out the fishing opportunities and the financial contribution provided for by the Fisheries Partnership Agreement between the European Community and the Republic of Seychelles. Official Journal L 345: 3-19.

174. European Union (2006) Protocol setting out the fishing opportunities and payments provided for in the Partnership Agreement between the European Community and Solomon Islands on fishing off Solomon Islands. Official Journal L 105: 39-53.

175. European Union (2010) Protocol setting out the fishing opportunities and financial contribution provided for in the Agreement between the European Union and Solomon Islands for the period from 9 October 2009 to 8 October 2012. Official Journal L 190: 9-26.
